# Microliter-scale *in vitro* aerosol-cell delivery of inhalable antisense oligonucleotide (ASO) induces “burst-like” pharmacokinetics^☆^

**DOI:** 10.1016/j.ijpx.2026.100588

**Published:** 2026-07-01

**Authors:** Pramod Kumar, Ashesh Chakraborty, Elias Richter, Lin Yang, Andreas Schröppel, Coralie Mergault, Larissa Rothkirch Née Knüppel, Frank Jaschinski, Marion Blayac, Rita Vanbever, Laurent Plantier, Nathalie Heuzé-Vourch, Otmar Schmid

**Affiliations:** a Institute of Lung Health and Immunity, Comprehensive Pneumology Center (CPC-M) with the CPC-M bioArchive, Ludwig-Maximilians-Universität München and Helmholtz Munich, Member of the German Center for Lung Research (DZL), Germany; b Centre d'Etudes des Pathologies Respiratoires / Inserm UMR1100, Tours, France; c Université de Tours, Tours, France; d Secarna Pharmaceuticals GmbH & Co. KG, Planegg, Germany; e Université catholique de Louvain, Louvain Drug Research Institute, Advanced Drug Delivery & Biomaterials, Belgium; f CHRU de Tours, Service de Pneumologie et Explorations Fonctionnelles Respiratoires, Tours, France

**Keywords:** Cloud MAX technology, Precision aerosol-cell delivery, Dosimetry, Idiopathic pulmonary fibrosis (IPF), Primary human lung fibroblasts, LIMCH1 gene silencing

## Abstract

Unlike traditional submerged cell cultures, nebulized drug delivery to air-liquid interface (ALI)-cultured lung cells provides a clinically relevant route for *in vitro* testing of inhalable drugs. Current nebulizer systems deliver non-physiologically large drug volumes. Here, we introduce the Cloud MAX technology, characterize its accuracy of dosimetry, and apply it to toxicity, efficacy, and biokinetics or permeability testing of a locked nucleic acid-modified antisense oligonucleotide (ASO). On average the Cloud MAX delivers 44% of 3–20 μL aerosolized liquid to ALI-cultured cells, the coefficient of variation (CV) of repeated drug dosing is <11%-rel. and as little as 1 μL of drug/insert (=0.25 μL/cm^2^) can be delivered to ALI-cultured cells. As proof of concept for dose-controlled *in vitro* drug testing, we demonstrated target engagement of a LIM and Calponin Homology domain 1 (LIMCH1)-specific ASO in primary lung fibroblasts from Idiopathic Pulmonary Fibrosis (IPF) patients “shielded” by an A549 alveolar epithelial layer mimicking inhalation therapy. LIMCH1-ASO was effective in LIMCH1-silencing even at the lowest dose (0.64 nmol/cm^2^). Nebulization did neither impair LIMCH1-ASO efficacy nor induce cytotoxicity for the highest tested dose (3.21 nmol/cm^2^). The permeability (biokinetics) of nebulized ASO through the A549 cell barrier was 14-fold faster (“burst-like”) than for larger volume (pipetted) ASO application. Computational modeling revealed that this well-known “burst-like” pharmaco−/biokinetics profile is due to small-volume drug application, not due to nebulizer-induced changes on barrier tightness (apparent permeability *P*_*app*_). Thus, the Cloud MAX is a substance-efficient platform for *in vitro* pharmacokinetics/−dynamics testing of inhalable drugs for targeted pulmonary therapy.

## Graphical abstract

**Figure:**
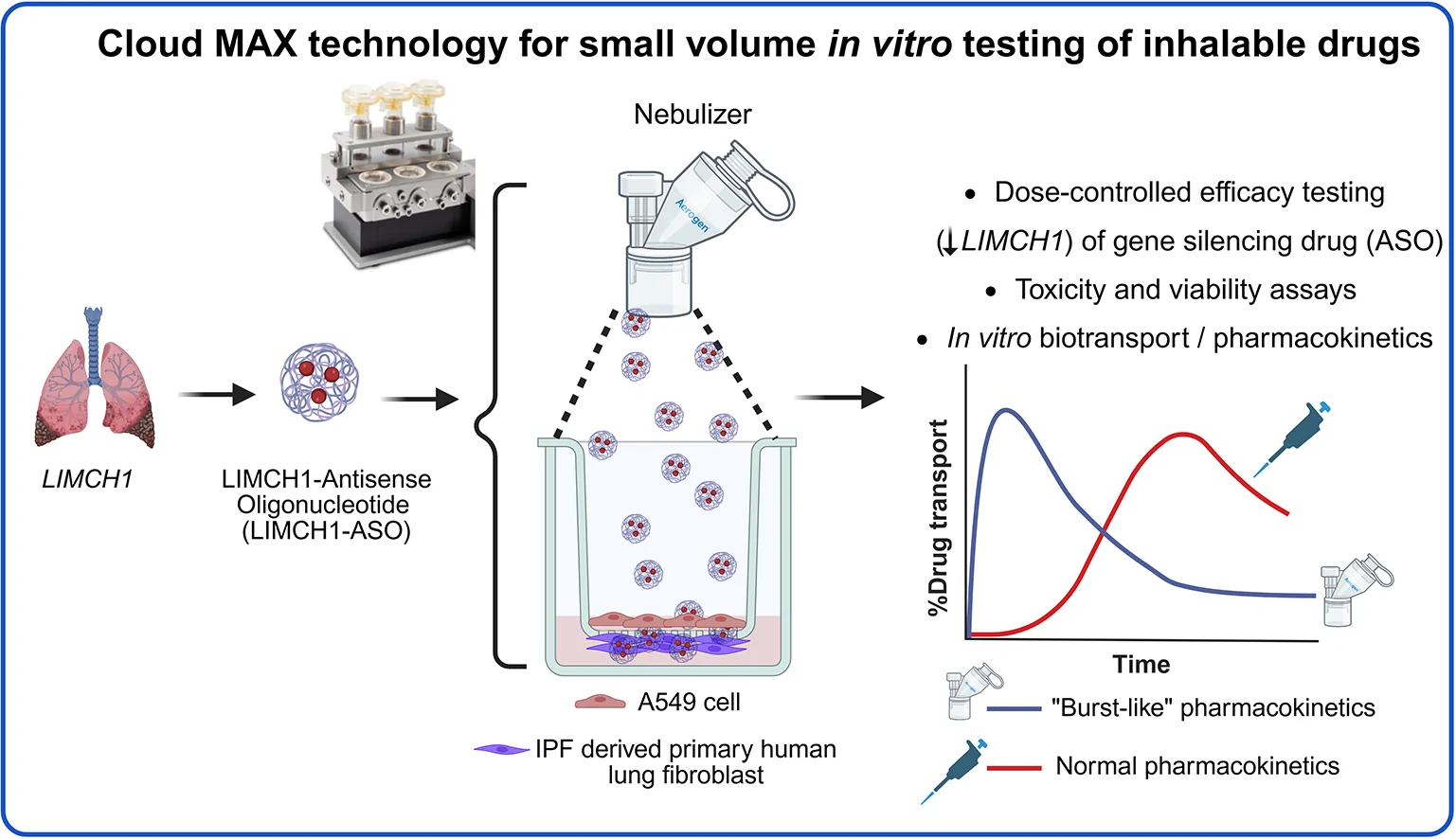

## 1 Introduction

Pulmonary aerosol delivery is a well-established route of drug administration for topical (targeted) inhalation therapy in chronic respiratory diseases such as asthma and chronic obstructive pulmonary disease (COPD). This approach is particularly suitable for treating lung disorders because delivering the drug directly to the target organ maximizes the therapeutic index compared with other administration routes (Yang et al., 2019; Yang et al., 2024). Unlike any other route of drug application, inhalation therapy enables direct deposition of a substantial fraction of the administered drug into the lungs, which maximizes the ratio of pulmonary to non-pulmonary drug dose throughout the organism. This localized drug delivery also reduces metabolic degradation of the drug *e.g.* in the gastrointestinal tract and liver. Taken together inhalation therapy enhances the therapeutic efficacy and minimizes off-target adverse effects for treatment of lung diseases (Hye et al., 2023; Yıldız-Peköz and Ehrhardt, 2020). Essential parameters for evaluating the suitability of drug candidates or devices for pulmonary delivery (or *in vitro* aerosol-cell delivery systems as introduced here) include assessments of aerosolization characteristics such as particle size distribution, pulmonary drug delivery efficiency (dosimetry), deterioration of integrity/efficacy of drug due to aerosolization, and potential toxic effects of the aerosolized drug to the lungs (Dabbagh et al., 2018). Physiological cell-based *in vitro* models that recapitulate the inhalation process and enable accurate quantification of cell- or tissue-specific delivered dose are considered crucial for preclinical assessment of toxicologic or therapeutic activity and subsequent *in vitro* to *in vivo*/clinical conversion of the *in vitro* results (Yıldız-Peköz and Ehrhardt, 2020; Ehrmann et al., 2020). Aerosolized drug delivery to respiratory epithelial cell layers cultured at air-liquid interface (ALI) conditions offers a physiologic model for inhalation therapy, potentially enhancing the predictive power of *in vitro* screening outcomes for clinical translation.

Over the past decades there has been substantial progress towards physiologic testing of inhalable drugs with *in vitro* models of the lung and aerosol-cell exposure systems. Historically, cell-based drug efficacy testing was performed with cells cultured under submerged cell culture conditions, where cells are cultured on the plastic dish filled with cell culture medium. While this setting may be physiologically relevant for most organs, it does not mimic the ALI condition encountered by lung epithelium during inhalation therapy. A key element of physiologic *in vitro* testing of inhalable drugs is the establishment of respiratory epithelial cultures under ALI conditions, which is critical for promoting polarization and differentiation, enabling epithelial cells to assume physiologic morphology and function, such as mucus or lining fluid secretion (Chakraborty et al., 2025; Stoleriu et al., 2024). Moreover, ALI culture conditions avoid inadvertent, non-physiologic interaction of the cell culture medium with the drug and allows direct aerosol deposition onto the apical side of the cells - mimicking the conditions in the lung during inhalation therapy (Lehmann et al., 2011; Darquenne et al., 2016; Paur et al., 2011; Primavessy et al., 2021). Depending on the drug, these lung “barrier” models may consist solely of epithelial cells or include co-cultures with disease-relevant cell types such as fibroblasts for pulmonary fibrosis or endothelial cells for pulmonary hypertension (Ainscough et al., 2022; Doryab et al., 2022). For enhanced clinical relevance some or all of these cell types can be primary cells extracted from healthy or diseased donors without immortalization. These physiologic cell culture models of the lung can be integrated into microfluidic “chips” or millifluidic bioreactors which mimic blood circulation and breathing-induced mechanoelastic effects on the alveolar tissue with medium perfusion and cyclic cell stretch, respectively (Doryab et al., 2022; Doryab et al., 2021; Huh et al., 2010). These complex models of the lung are often referred to as lung-on-a-chip or mini-lung, however, all of the current *in vitro* cell models only reflect a small subset of the more than 50 types of lung cells and often do not adequately reflect the 3D microenvironment of the lung (Travaglini et al., 2020). Thus, careful selection of the most relevant cell types for addressing a specific research question is essential for obtaining clinically relevant results from *in vitro* cell culture experiments.

Nevertheless, combined with aerosolized drug-delivery methodologies, these physiologic *in vitro* systems hold strong potential for improving the predictive value of *in vitro* screening systems with respect to the clinical efficacy of new drugs (Ehrmann et al., 2020; Darquenne et al., 2016). These types of advanced *in vitro* test systems are also discussed in the context of replacement, reduction, and refinement of animal testing (3R principle) for the identification and approval of new chemicals under the European REACH (registration, evaluation, authorization, and restriction of chemicals) framework. One of the common hallmarks of many chronic lung diseases, including asthma, COPD, and idiopathic pulmonary fibrosis (IPF) are characterized by persistent inflammation and progressive tissue remodelling. Understanding the underlying disease mechanisms and identifying molecular targets for therapeutic intervention is often very difficult, and currently no cure exists for any of the chronic lung diseases. Recent technological advancements in high-throughput transcriptomics and single-cell sequencing on a whole-organ level in small animal models have identified numerous promising cell type-specific molecular targets for various lung diseases (Aldridge and Teichmann, 2020). For instance, there is evidence that the LIM and calponin homology domains 1 (LIMCH1) is upregulated in fibroblasts and myofibroblasts in both IPF diseased patients' lung (http://www.ipfcellatlas.com/) (Reyfman et al., 2019) and bleomycin-treated mouse model of IPF (Fig. S1) (Strunz et al., 2020). Addressing the underlying genetic and molecular contributors to these disease conditions has become central to the development of advanced therapies. Inhaled oligonucleotides have gained significant interest in this context, particularly as part of gene therapy approaches. In particular, antisense oligonucleotide (ASO) therapeutics have emerged as a promising strategy, offering substantial potential to modulate disease pathways at the mRNA level (Liao et al., 2017). This growing interest in ASO-based treatments has been encouraged by the FDA approval of several therapies, including nusinersen for spinal muscular atrophy and inotersen for treatment of patients with hereditary transthyretin amyloidosis, however all FDA-approved ASO therapies use earlier generation chemistries (1^st^ and 2^nd^ generation) (Egli and Manoharan, 2023; Martinovich et al., 2018). In this project, ASOs were used that contain locked nucleic acid (LNA)-modified nucleotides in the flanks, a chemical modification that greatly enhance affinity to the target RNA resulting in higher efficacy and potency thereby strongly increasing the therapeutic window (Wahlestedt et al., 2000). Furthermore, ASOs using this type of chemical modification do not require transfection or delivery reagents for activity *in vitro* and *in vivo*. Efficient target knockdown can be achieved by application of unformulated/naked ASOs in a variety of cell types and tissues *in vitro* and *in vivo* (Stein et al., 2009).

The development of inhalable biologics for therapeutic intervention (*e.g.*, antibodies, proteins, siRNA, and ASO) involves a sequence of preclinical studies to assess the efficacy, target specificity, and toxicity of the drug candidates. Typically, these studies begin with *in vitro* cell culture models followed by *in vivo* animal experiments. In current drug testing programs, aerosolization is typically addressed during device selection, which usually occurs late in the preclinical phase, close to entry into clinical development. Although lung epithelial models cultured under physiologic ALI conditions may provide more clinically predictive results, including burst-like pharmacokinetics, cell-based assays are still largely absent from preclinical drug testing of aerosolized drugs (Lee et al., 2023). The implementation of aerosolized drug delivery in early preclinical drug development is not only relevant from the biological perspective (drug delivery to ALI cell culture models of the lung - see above), it is also essential from the perspective of the drug compound, since the aerosolization process can impair the molecular structure and hence efficacy/toxicity profile of drugs, especially if they have a complex molecular structure such as biotherapeutics (Darquenne et al., 2016).

The methodologies for cell-based *in vitro* testing of nebulized drugs with respect to their stability, toxicity and efficacy are still relatively limited. Many existing aerosol-cell exposure systems, which were designed for acute inhaled ambient or occupational particle toxicity studies are not suitable for inhalable drug testing albeit the often used clinically relevant orifice or vibrating mesh nebulizers. The aerosol cell exposure system is based on continuous flow system, where a continuous aerosol flow is guided over the ALI-cultured cells through gravitational settling and only a small fraction of the invested dose is deposited on the cells (Paur et al., 2011). For instance, therapeutic aerosols with a median aerodynamic diameter of 2–5 μm exhibit relatively slow gravitational settling, necessitating low airflow rates to achieve efficient deposition, whereas higher airflow rates result in significantly reduced deposition efficiency. Consequently, it would require days or weeks of continuous exposure to reach clinically relevant doses (Paur et al., 2011). One of the few exceptions is the ALICE Cloud technology equipped with a clinically relevant vibrating mesh nebulizer (Aeroneb Lab/Pro; Aerogen Inc., Galway, UK) which can generate a very dense cloud of aerosol - without using any carrier air - by air flow-free nebulization into a closed exposure chamber with ALI cultured cells placed at the bottom of the chamber, which was initially developed for particle toxicity assessment (Lenz et al., 2009) and subsequently adapted for drug efficacy studies (Lenz et al., 2014; Ritter et al., 2023). Yet, this technology is currently limited to drug delivery efficiencies of *ca.* 15% (in the best case) (Lenz et al., 2014) and it requires a minimum nebulization volume of *ca.* 200 μL. Considering the large number of *in vitro* test settings, this may lead to challenges with respect to the amount of drug and cost, especially for expensive experimental drugs involving biologics. Therefore, we developed the (ALICE) Cloud MAX technology, which is a refined version of the ALICE Cloud technology requiring as little as 3 μL of drug and yielding a higher drug delivery efficiency than the ALICE Cloud technology. Both the ALICE Cloud and the (ALICE) Cloud MAX are commercially available as VITROCELL Cloud (Alpha) and VITROCELL Cloud (Alpha) MAX, respectively (VITROCELL Systems, Waldkirch, Germany).

As a proof of concept for the Cloud MAX technology in preclinical drug testing with novel biotherapeutics, we evaluated the toxicity, deposition efficiency, biotransport kinetics, and target engagement (efficacy) of LNA-modified LIMCH1-specific ASO delivered using a clinically relevant Aeroneb Solo nebulizer to an alveolar cell barrier model comprising primary human lung fibroblasts derived from IPF patients (IPF-HLFs) co-cultured with A549 alveolar epithelial cells under ALI conditions. Quantitative dosimetry of nebulized fluorescein and fluorescently labelled (Cy5)-ASO on and across the epithelial barrier (biokinetics) was determined for nebulized volumes ranging from 3 to 20 μL. LIMCH1-ASO efficacy at non-toxic doses was evaluated by quantifying downregulation of human *LIMCH1* mRNA and protein levels in primary human lung fibroblasts and the underlying mechanisms of burst-like pharmacokinetics (here biokinetics) were investigated.

## 2 Materials and methods

### 2.1 Cloud MAX design and operation

VITROCELL Systems has recently introduced a commercial nebulization device into the market named as VITROCELL® Cloud Alpha MAX. The prototype of this device (Fig. 1A), the ALICE Cloud MAX, was designed and assembled inhouse at Helmholtz Munich (Munich, Germany). The ALICE Cloud MAX was designed for dose-controlled and substance efficient aerosol exposure on cells cultured on 6-well transwell inserts under ALI condition. The commercial versions of the Cloud MAX technology, VITROCELL® Cloud Alpha MAX of the first generation and recent Cloud Alpha MAX in the double throughput version are depicted in Fig. 1B and C. The core element of the Cloud MAX technology is an exposure manifold with six exposure units - one for each of the six transwell inserts. Each exposure unit consists of a cylindrical exposure manifold, a nebulizer, and an adaptor that connects a clinically used vibrating mesh nebulizer (see below for details) to the exposure manifold. Three versions of the exposure manifold are available, which vary only in the height of the exposure tube (20, 40, or 80 mm; here: 20 and 40 mm are used), while the inner diameter remains constant at 25 mm, ensuring that a cylindrical aerosol cloud forms directly above the cell-covered surface of each transwell insert. Standard transwell inserts from BD (total area of 4.7 cm^2^ with 4.2 cm^2^ cell growth area, PET membrane with 0.4 μm pore size, Catalog number 353090, Becton Dickson (BD) Labware, USA) with tapered geometry (wider on top than bottom of the insert) and closed lateral walls preventing unintentional aerosol exposure to the basolateral side of the insert containing cell culture medium. For transwell inserts with partially open lateral walls (*e.g.*, from Corning Inc.), it is recommended that the medium level in the basal compartment be reduced slightly, so that any drug reaching the medium through the partially open lateral walls cannot come in contact with cells from the basal side. Typically, cells will tolerate incubation for *ca.* 7.5 min (duration of aerosol-cell exposure process - see below) without direct contact to the medium. Yet, if this should be a problem, a small amount of medium (*ca.* 25 μL/cm^2^, *i.e.*, *ca.* 100 μL per 6-well insert) could be pipetted on top of the inserts. If medium contact is necessary for the cells during the exposure period, VITROCELL Systems provides “media covers” to prevent inadvertent direct aerosol deposition into the basal medium.

**Fig. 1:**
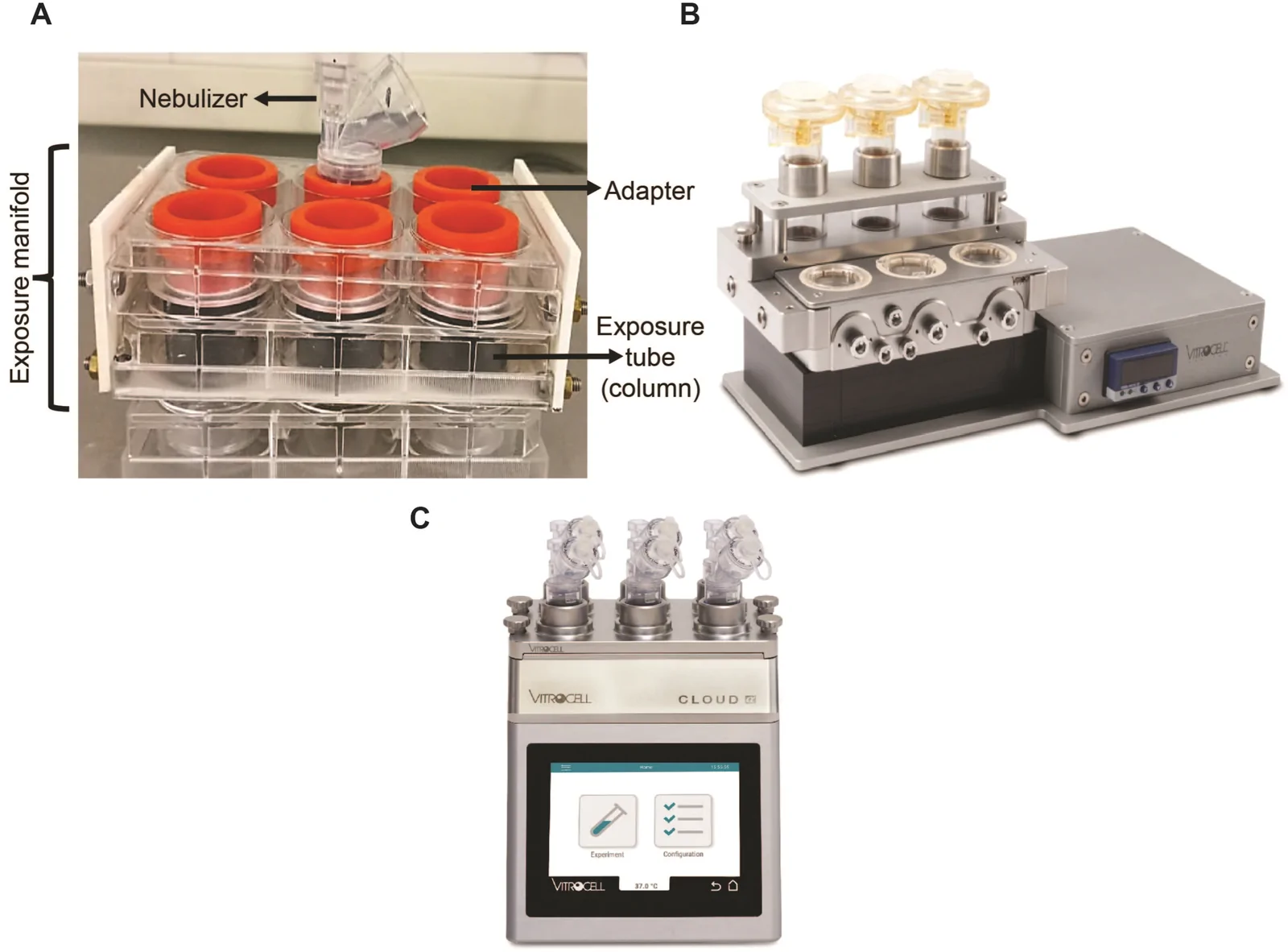
Depiction of the Cloud MAX aerosol-cell exposure systems. (A) Depiction of the prototype version of the Cloud MAX device depicting the main components namely cylindrical exposure tubes (units of 3 or 6 tubes are referred to as exposure manifold), an airless vibrating mesh nebulizer and an adaptor connecting the nebulizer to the exposure tube. The nebulizer gradually” fills” the exposure tube with an aerosol cloud which gradually settles onto the 6-well transwell insert placed at the bottom of each exposure tube. (B) Photo of a commercially available VITROCELL® Cloud MAX of the first generation and (C) the currently available VITROCELL® Cloud Alpha MAX. The length of the commercially available exposure tubes can be 20, 40, or 80 mm.

The aerosolization procedure requires filling the reservoir volume of a nebulizer with liquid and then connect the nebulizer to the exposure tube *via* an adaptor. Here, the widely used Aeroneb Lab/Pro/Solo vibrating mesh nebulizers (Aerogen Inc., Galway, Ireland) operated with an Aeroneb® Lab Control Module (Catalog number: NEB7000, Kent Scientific, USA) were used for generating droplets with a volume median aerodynamic diameter (VMAD) of 2.5–6.0 μm. The 6-well exposure manifold was designed to fit directly onto a 6-well plate containing transwell inserts (Fig. 1A). Since the proximity of adjacent exposure tubes does not allow for simultaneous operation of six nebulizers, the same nebulizer was used sequentially for all exposure units. Each nebulization of 3 to 10 μL liquid requires less than 5 s followed by a 55 s cloud settling period after which the nebulizer was removed slowly from its position, placed onto the next exposure tube and refilled for the next exposure. This process was repeated until all exposure tubes were filled with aerosol and an additional 1 min settling time was granted for the last exposure tube to guarantee complete gravitational settling of the aerosol cloud onto cells. The total exposure time for the aerosolization procedure across 6 transwell inserts was 7.5 min, which includes a few seconds for refilling the nebulizer with up to 10 μL of liquid prior to starting the next nebulization. Unless stated otherwise, all aerosol-cell exposures (or performance testing) were performed with the ALICE Cloud MAX with 20 μL of nebulized substance and a 40 mm column height.

### 2.2 Antisense oligonucleotide

LIMCH1-specific ASO and non-targeting control oligonucleotide were identified by Secarna Pharmaceuticals GmbH & Co. KG and synthesized by Axolabs GmbH, Kulmbach. Sulfo-cyanine 5 labeled LIMCH1-ASO was purchased from AxoLabs (Kulmach, Germany). The dye (SulfoCy5-NHS) was linked to the ASO 5′ end *via* an aminohexyl linker. The sequences used for non-targeting oligonucleotide and LIMCH1-ASO are listed in Table 1.

**Table 1:** Sequences used for Non targeting oligonucleotide and LIMCH1-ASO

Target	Sequences
Non targeting oligonucleotide	+C* + G* + T*T*T*A*G*G*C*T*A*T*G*T*A* + C* + T* + T
LIMCH1-ASO	+G* + G* + A*T*T*C*G*A*G*A*G*T*A*G* + T* + T* + T

+ indicates LNA-modified nucleotides and *indicates Phosphothioate Oligonucleotide linkages.

Non-targeting control oligonucleotide (Molecular weight: 5.96 kDa) and LIMCH1-ASO (Molecular weight: 5.70 kDa) were dissolved in 5% glucose solution to achieve a stock concentration of 10 mg/mL. For the two different working concentration of non-targeting control oligonucleotide and LIMCH1-ASO used in this study, the stock solution was further diluted in 5% glucose solution to achieve a working concentration of 3 nmol (0.64 nmol/cm^2^) and 15 nmol (3.21 nmol/cm^2^).

### 2.3 Reagents and materials for cell culture experiments

All chemicals and reagents used were of analytical grade and purchased from Sigma-Aldrich Germany, unless stated otherwise. QuantiGene Singleplex RNA and Luciferase assay Reagent ONE GlO™ were procured from Thermo Fischer Scientific Germany and Promega Corp., Madison, USA (Catalog number: QGS-200 and E6110, respectively).

### 2.4 Cell culture

In this study, several *in vitro* models were employed to evaluate the toxicity, target engagement, and biokinetics of LIMCH1-ASO. The models included monocultures of alveolar type II-like epithelial cells (A549), IPF patient-derived fibroblasts (IPF-HLFs), a coculture model comprising both cell types (A549 and IPF-HLFs), and a human fibroblast cell line (CCD-16Lu). IPF-HLFs were obtained from the CPC-M bioArchive at the Comprehensive Pneumology Center (CPC-M, Germany) (*n* = 3). The study was approved by the local ethics committee of the Ludwig-Maximilians Universität München, Germany (Ethic vote #19–630). Written informed consent was obtained from all study participants. IPF-HLFs were expanded in a DMEM/F-12 medium with 1% Pen/Strep (Catalog number: 15140–122, Gibco), 10% Fetal Bovine Serum (FBS) (Catalog number: S0615, Sigma-Aldrich) and incubated in a humidified cell incubator with 95% air and 5% CO_2_ at 37 °C. A549 cells were used either provided by ATCC (ATCC CCL-185) or stably transfected with a luciferase reporter gene under the control of the promotor of the pro-inflammatory interleukin 8 (IL8) (A549-Luc-IL8) (courtesy of A. Duschl, Paris Lodron University of Salzburg, Austria (Oostingh et al., 2008). A certain volume of fluorescein sodium (CAS 518–47-8) solution (15 μg/mL in PBS; fluorescein sodium serves as surrogate drug) was nebulized into each exposure tube using an Aeroneb Lab nebulizer on 6 well transwell insert pre-filled with 1 mL of PBS on the apical compartment. After nebulization, the PBS from the apical compartment was collected, loaded on the 96 well plates with a transparent bottom (Catalog number: 655086; Greiner Bio-One GmbH Germany), and then the fluorescent intensity was determined using a fluorescence microplate reader (Tecan Safire II, Tecan Inc) at an excitation/emission wavelength of 483/525 nm. Using a standard curve, the fluorescent intensity obtained which was then converted to the fractional deposited aerosol efficiency for each exposure tube. The method provides insights about the drug delivery performance with respect to spatial uniformity and repeatability, insert-to-insert dose variability and their dependence on nebulized liquid volume (3–10 μL; completely nebulized), length of the exposure tubes (20 mm, 40 mm), and Cloud MAX system. Prior to LIMCH1-ASO treatment, the fluorescein-based aerosol deposition characteristics was compared to that of fluorescence (Cy5) labeled LIMCH1 ASO (Cy5-ASO) at a wavelength of 685 nm to ensure proper performance of the Cloud MAX for delivery of our active drug (LIMCH1-ASO).

Fluorescence imaging of nebulized fluorescent nanoparticles was used to determine the distribution of a nebulized substance across the membrane covered with cells in a 6-well transwell inserts. For this, 5 μL of an aqueous SkyBlue suspension (10 mg/mL fluorescent polystyrene particles; diameter: 0.51 μm; Kisker Biotech GmbH & Co. KG, Steinfurt, Germany, 1:3 dilution in PBS) was filled into the nebulizer and completely nebulized on the transwell inserts using the ALICE Cloud MAX device with 20 mm exposure tube. Subsequently, the individual components of the system were fluorescence imaged using *in vivo* imaging system (IVIS; Lumina II, PerkinElmer) with an excitation filter 640 nm and emission filter Cy5.5. To measure the aerosol dose deposited in the non-transparent adapter, the adaptor was lined with a white tissue tape, which was then removed, and the fluorescence intensity was determined on the white tissue tap.

### 2.5 LIMCH1- ASO nebulization using the Cloud MAX device

For aerosolized delivery of LIMCH1-ASO to ALI cultured cell models, 20 μL of LIMCH1-ASO suspension was nebulized with Aeroneb Solo nebulizer (Aerogen Inc., Galway, Ireland) positioned on the ALICE Cloud MAX with 40 mm exposure tubes. The effect of ALICE Cloud MAX mediated nebulization (20 μL saline; 0.9% NaCl) on A549-Luc-IL8 cells was determined as described in Lenz AG et al., study (Bannuscher et al., 2022; Lenz et al., 2014). For this purpose, a ‘worst-case’ configuration of the Cloud MAX device with respect to aerosol impact on the cells was used, in which the nebulizer was placed directly above the transwell insert (0 mm exposure tube length), with an approximate distance of 30 mm between them. This configuration results in the highest possible adverse effect from direct aerosol impaction onto the cells, while all other cell-handling characteristics remained unchanged.

### 2.6 LIMCH1-ASO drug delivery *in vitro*

The primary IPF-HLFs monoculture or the A549-IPF-HLFs coculture models used for ASO exposure were grown to confluency as described above and subsequently starved for 24 h (0.5% FBS, instead of 10% FBS) followed by 24 h acclimatization phase prior to the application of 3 nmol and 15 nmol LIMCH1 ASO or non-targeting control oligonucleotide on the apical side (*i.e.*, 0.67 or 3.21 nmol/cm^2^). The LIMCH1-ASO was applied either *via* nebulization as described above (7 μL per insert; 430 μM or 2150 μM ASO solution) or by pipetting of 100 μL of non-nebulized LIMCH1-ASO solution (30 and 150 μM ASO) on the air-lifted apical side of the cell culture models. The cells were then incubated for 3 days followed by replacing 100 μL basal culture medium with fresh medium, and cells were further incubated for 4 days at the ALI condition. Non-targeting control oligonucleotide was used as a negative control.

For LIMCH1-ASO drug delivery to CCD-16Lu cells cultured under submerged conditions, 500 μL of an 84 μM ASO solution (either non-targeting control oligonucleotide or LIMCH1-specific) was aerosolized using an Aeroneb Solo mesh nebulizer and collected in a 15 mL tube. CCD-16Lu cells were then cultured in 1 mL of the nebulized ASO solution diluted in DMEM-GlutaMAX w/o FBS to achieve a final ASO concentration of 1 μM (0.26 nmol/cm^2^) and incubated for 72 h. For assessment of the effect of nebulization on ASO efficacy, 500 μL of an 84 μM ASO (control or LIMCH1-specific) solution was aerosolized using the Aeroneb Solo mesh nebulizer and collected in a 15 mL tube. After 24 h serum deprivation, submerged-cultured CCD-16Lu fibroblasts were incubated with 1 mL of the nebulized or non-nebulized (pipetted) ASO solution diluted in DMEM-Glutamax w/o FBS to a final concentration of 1 μM (*i.e.*,0.29 nmol/cm^2^) for additional 72 h.

### 2.7 Luciferase assay to estimate Interleukin 8 (IL 8) activity

To test the nebulization itself has an impact in inducing inflammatory response on cells, A549-Luc-IL8 cells were used to evaluate the IL8 protein levels following exposure to 10 μL saline (0.9% NaCl) nebulization. After 24 h nebulization, the cells were lysed by adding lysis buffer (Catalog number: E2661, Glo Lysis Buffer Promega Corp., Madison, USA) and incubated on ice for 5 min. The cell lysates were transferred into a 1.5 mL Eppendorf tube and centrifuged at 350*g* for 1 min at 4 °C. The supernatant was collected, diluted with lysis buffer (1:2 dilution) and mixed further with equal volume of luciferase assay reagent in a 96 well plate with a transparent bottom. The emitted light from the bioluminescent reaction was measured using a luminometer at a wavelength of 540 nm. As a positive control for maximal IL8 release, A549 cells were cultured under submerged condition and were treated with TNFα (15 ng/mL) *via* the basolateral compartment of the insert for 24 h.

### 2.8 Cell viability

#### 2.8.1 WST-1 assay

After 24 h post LIMCH1-ASO delivery *in vitro*, the cell viability was determined using a colorimetric metabolic activity assay (Water-Soluble Tetrazolium 1 (WST1), 11644807001, Roche, Mannheim, Germany) according to the manufacturer's instructions. The WST1 reagent was diluted with DMEM/F-12 medium without FBS (1:12 dilution, 1 mL) pipetted on the apical side of the insert and incubated on cells for 10 min. Subsequently, 500 μL of the incubated WST1 solution was transferred from the apical side of the insert to a 96 well plate in three technical replicates of 100 μL each, and the absorbance was measured at 450 nm using a fluorescence microplate reader. The cell viability of A549-Luc-IL-8 cells exposed to 10 μL 0.9% NaCl nebulization was determined using a colorimetric metabolic activity assay (WST1, Roche, Mannheim, Germany).

#### 2.8.2 Lactate dehydrogenase assay

As measure of cytotoxicity experienced by cells due to the exposure to LIMCH1-ASO delivery, supernatant from the basal compartment of the insert was collect, and release of lactate dehydrogenase (LDH) was determined using the cytotoxicity detection LDH kit (Roche, 11644793001, Mannheim, Germany) according to the manufacturer's instructions. The positive control used for maximal LDH release was achieved by collecting supernatant from cells lysed with 1% Triton X-100 in PBS.

#### 2.8.3 3-(4,5-Dimethylthiazol-2-yl)-5-(3-carboxymethoxyphenyl)-2-(4-sulfophenyl)-2*H*-tetra-zolium (MTS) assay

CCD-16Lu cells were seeded (5000 cells/well) in a 96-well plate and cultured for 4 days in DMEM-GlutaMAX with 1% FBS and were then treated with either non-targeting control oligonucleotide or LIMCH1 ASO. Upon treatment, the cell metabolic activity was assessed with the MTS reagent (Catalog number: G3580, Celltiter, Promega) as a marker for cell viability and was performed according to the manufacturer's instructions.

### 2.9 Transport kinetics and apparent permeability of Cy5 tagged ASO (Cy5-ASO) across an alveolar epithelial cell model

Here, an A549 monoculture *in vitro* model was used to study the dose “shielding” effect of the alveolar epithelium on the fibroblasts as effector cells, which are located in the interstitial tissue of the alveolar region in close proximity to alveolar type II cells. By culturing the A549 cells on the apical side of the insert, the transport of Cy5-labelled ASO (Cy5-ASO: phosphodiester linked, 0.85 Da) across the A549 epithelium cell barrier into the basolateral compartment of the insert (containing 1.2 mL phenol free DMEM/F12 medium) can be monitored representing the Cy5-ASO transport rate to the fibroblasts located on the basal side of the insert (Figs. 5A, 6A - bottom). A dose of 18.4 μg of Cy5-ASO (3 nmol, molar mass of ASO 6151 Da) was applied apically on the cell barrier either by nebulization (7 μL was delivered after 20 μL nebulization using the Aeroneb Solo nebulizer; see Fig. 4) or as pipetted liquid (100 μL) and incubated for varying periods of time. During the *ca.* 2 min of nebulization in the Cloud MAX, the insert was not in contact with the basal medium to avoid inadvertent loss of Cy5-ASO prior to end of the aerosol exposure. After the exposure, the insert was placed in a 6-well with fresh 1.2 mL medium and Cy5-ASO transport into the basal medium starts. No such precautionary measures were necessary for pipetted application, since pipetting occurs instantaneous. For obtaining a biokinetic profile, a 100 μL aliquot of the basal medium was collected at various time points ranging between 0.25 h and 72 h and was further replenished with an equal volume of fresh medium followed by mixing. To determine the apical fraction of non-transported Cy5-ASO, after 72 h incubation, the remaining apical ASO was collected using gentle PBS washing. Moreover, the Cy5-ASO fraction associated with the A549 cells was determined by lysing cells using three repetitive liquid nitrogen freeze-thaw cycles and subsequently collecting the cell lysates in 1 mL PBS. At the end, the Cy5-ASO fractions associated with the apical, cell (lysate), and basal compartments were determined using a fluorescence microplate reader at the wavelength of excitation/ emission wavelength of 649/667 nm.

#### 2.9.1 Calculation of apparent permeability

For quantitative assessment of the biokinetics data, the widely used concept of apparent permeability *(P*_*app*_*)* was used. The apparent permeability coefficient (*P*_*app*_, cm/s) was calculated with Eq. (1a) (Jorgensen et al., 2025).

(1a) $$P_{\mathrm{app}}=\frac{\mathrm{dQ}}{\mathrm{dt}}\;\frac{1}{AC_{0}}$$

where *dQ/dt* is the average molar transport rate of Cy5-ASO (mol/s), which can be calculated from *dC(t)* × *V*_*basal*_*/dt*, *i.e.* the measured change in concentration (mol/mL) and medium volume (mL) in the basal compartment during the time interval *dt*. Constants *A* and *C*_*0*_ represent the surface area of the insert membrane (4.67 cm^2^) and the initial concentration of Cy5-ASO in the apical compartment (mg/mL; here: nmol/mL), respectively. Eq. (1a) can be rewritten by considering that *dQ = dD* (change in molar dose in the basal compartment) and *C*_*0*_ *= D*_*mol,0*_*/V*_*apical*_ yielding

(1b) $$P_{\mathrm{app}}=\frac{\mathrm{dD}{\left(\%\right)}_{\mathrm{mol}}}{\mathrm{dt}}\;\frac{V_{\mathrm{apical}}}{A}$$

where *dD(%)*_*mol*_ *(= dD D*_*mol,0*_^*−1*^*)* is the relative change in dose (in mol) in the basal compartment. Eq. (1b) is algebraically identical to Eq. (1a), but it will prove instructive for understanding the effect of *C*_*0*_ on the transport rate across the cell-membrane barrier. In this context, it is important to note, that *D*_*mol,0*_ represents the total molar dose in the transwell system independent of time, *i.e.* the sum of the drug dose in the apical, basal, and cell-membrane compartment at any given time (in the absence of wall losses).

#### 2.9.2 Exponential curve fit of biokinetics profile for nebulized application

It is important to note that Eqs. (1a), (1b) are valid only for approximately constant *C*_*0*_ *i.e.*, for low transported dose fraction, typically less than *ca.* 25% of the initially applied dose. This condition was met for pipetted application (23.1% ± 2.1% at 0.25 h), but not for ASO delivery (75.4 ± 6.7% at 0.25 h). For estimation of the required time for reaching 23.1% dose level after nebulization or pipetting, an exponential curve fit of the time-dependent basal ASO dose was performed yielding Dose_neb_(%) = 83.6% * [1–exp(−b*t)], with b (h^−1^) = 9.662 (95% CL: 7.050–21.41). Due to the lack of data between zero and the plateau value, the uncertainty in b is relatively large and the fitting routine was forced through the origin and the plateau value of 83.6, since all data points up to 4 h were not statistically significantly different from this value (Fig. 5D).

### 2.10 Computational ASO transport model across an *in vitro* cell barrier

For a more in-depth understanding of the processes governing the observed fast “burst-like” biokinetics for nebulized ASO delivery, a computational model was setup which solves the transport equation for the transwell insert biokinetics assay. For simplicity, we converted the radially symmetric three-dimensional (3D) geometry of the transwell insert biokinetics assay into an analogous 1D geometry and computationally solved the 1D time-dependent diffusion transport equation given by

(2) $$\frac{\partial C\left(x,t\right)}{\partial t}=\mathrm{Diff}\frac{\partial^{2}C\left(x,t\right)}{{\partial x}^{2}},$$

where *C(x, t)* is the ASO concentration at position *x* and time *t* and *Diff* is the ASO diffusion coefficient (cm^2^/s). The details of this approach are presented in the supporting information. Briefly, the selected 1D geometry approximates the conditions along the centerline of the 3D geometry (Fig. S6A). As seen from the supporting information, the ASO diffusion coefficient *Diff*_*1D*_ *o*f 1.68 × 10^−6^ cm^2^/s in water was estimated from the molecular weight of the Cy5-ASO (6.1 kDa) and the Einstein-Stokes and van-der-Waal volume equations (see Supplementary Material). For reasons presented in the discussion Section 4.2 we note that the “effective” diffusion coefficient is 2.5-fold lower, namely 0.66 × 10^−6^ cm^2^/s.

From the diffusion coefficient *P*_*app*_ can be calculated from

(3) $$P_{\mathrm{app}}=\frac{\mathrm{Diff}}{h}$$

where the thickness *h* of the A549 cell barrier was assumed to be 10 μm (Ren et al., 2016). The initial and boundary conditions of the modeling are specified in the supporting information. The 1D transport equation was numerically solved using the built-in ode45 solver of MATLAB (version 2025a).

### 2.11 Immunofluorescence analysis

For immunofluorescent staining, the coculture *in vitro* model comprising of A549-IPF-HLFs was cultured on transwell inserts (Catalog number: 3452, Corning) with similar culture conditions as mentioned before for enhanced visibility for microscopy application. Initially, cells were washed three times for 5 min each with PBS at both apical and basal side of the insert. After washing, cells were fixed with 3% paraformaldehyde in PBS for 15 min at room temperature. The cells were then washed again with PBS and stored in 0.1 M glycine in PBS for 24 h at 4 °C. After incubation, the glycine was removed, and 0.2% Triton X-100 in PBS was added for permeabilization for 15 min. After permeabilization, cells were washed again with PBS for three times 5 min each at both apical and basal side of the insert and the cells were then incubated with Alexa Fluor 488 Phalloidin (1:400, Invitrogen, cat. A12379) and DAPI (4′,6-diamidine-2-phenylindole; 1:1000) to stain F- actin cytoskeleton and nucleus, respectively. The membrane of the insert containing cells were cut out, placed on the slide, and fixed with a coverslip using the fluorescent mounting medium (Dako, Denmark). The immunofluorescent staining images were obtained using Confocal fluorescence microscope (Zeiss) under 20× magnification.

### 2.12 bDNA, RNA isolation and qRT-PCR analysisp

For monoculture derived from only IPF-HLFs or the coculture from A549-IPF-HLFs cells, cells were first washed with PBS on both apical and basal side of the insert followed by collecting IPF-HLFs by scraping into QuantiGene™ Lysis Mixture (QP0524, containing proteinase K). In case of coculture model, A549 cells were carefully removed from the apical side and the IPF-HLFs at the basal side of the insert were collected into QuantiGene™ Lysis Mixture. The target mRNA level was determined using bDNA (branched DNA) assay (QuantiGene SinglePlex Assay Kit 96-Well plate format and QuantiGene Sample Processing Kit for cultured cells, Thermo Fisher Scientific) and was used according to manufacturer's instructions. The following probe sets were used: human LIMCH1 (SA-3006419); human HPRT1 (SA-10030). *LMICH1* mRNA expression levels were normalized to *HRPT1* as housekeeping gene. For CCD-16Lu cells, the RNA was extracted using the Nucleo Spin RNA XS (Catalog number: 740902.50, Macherey Nagel). mRNA was then reverse transcribed into cDNA using the First Strand cDNA Synthesis Kit (Thermofisher Scientific). Human *LIMCH1* transcripts (forward primer: GGTCAGTCACCAAACAGGTCT; reverse primer: GTCCCACTCACTGCATCTCC) were quantified by qRT-PCR (Sybr select Master Mix, Thermofisher) and expressed relative to human *UBC* (forward primer: CAGTTGGTCCTGCGCTTG; reverse primer: TTTTTTGGGAATGCAACAACTTT) as an endogenous control using the 2^-ΔΔCt^ method.

### 2.13 Protein isolation and western blot analysis

After LIMCH1 ASO nebulization on the co-culture A549-IPF-HLFs, A549 epithelial cells from the apical side of the insert were carefully removed by gentle scraping and IPF-HLFs were lysed using RIPA buffer (50 mM Tris·HCl, pH 7.6; 150 mM NaCl; 1% Nonidet P-40; 0.5% sodium deoxycholate; and 0.1% SDS) with protease inhibitor cocktail (05892970001, Roche, Basel, Switzerland) and PhosSTOP phosphatase inhibitor cocktail (PHOSS-RO, Roche). The cell lysates were collected and centrifuged at 14000 RPM for 5 min at 4 °C. The supernatant was collected and stored at -80 °C for further use. The protein concentration was determined using Biorad protein assay kit (Catalog number: 5000002) and used according to manufacturer instructions. Approximately, 25 μg of protein sample was mixed with 1× lamellae buffer (65 mM Tris-HCl, pH 6.8; 10% glycerol; 2% SDS; 0.01% bromophenol blue; and 100 mM DTT) and were separated in 10% SDS-PAGE gel. Proteins were transferred from SDS-PAGE gel to polyvinylidene difluoride (PVDF) membrane (Thermo Scientific, 88518, Rockford, IL) using a wet tank blotting system (Mini PROTEAN Tetra Cell, 552BR, Bio-Rad, Munich, Germany). The membrane blocking was carried out using 5% milk powder dissolved in 1× tris buffer followed by washing the membrane three times 10 min each with 1× tris buffer and incubated with LIMCH1 primary antibody (dilution 1:2500 in 5% milk powder dissolved in 1× tris buffer) (Catalog number: NBP2–39006, rabbit antihuman; NovusBio, USA) at 4 °C overnight. Next day, the membrane was washed three times 10 min each with 1× tris buffer and incubated with secondary antibody HRP conjugated anti-rabbit (GE healthcare) in 5% milk powder in 1× tris buffer at a dilution of 1:30000 for 1 h in room temperature followed by washing the membrane for three times 10 min each with 1× tris buffer. The membrane was visualized using Femto substrate (Catalog number: 34095, Thermofisher Scientific) and analyzed using Bio-Rad scanner. Peroxidase conjugated beta actin antibody (1:40000, 1 h at room temperature) was used as a house keeping protein for normalization.

Post LIMCH1-ASO on CCD-16Lu fibroblast cells, the total cell proteins were recovered in 100 μL lysis buffer (50 mM Tris HCl pH 7.4, 150 mM NaCl, 0.1% SDS, 0.5% sodium deoxycholate, 1% Triton X-100) with 1% protease and phosphatase inhibitor (PPC1010, Sigma) and was quantitated using Bicinchoninic acid Assay (Pierce, Thermofisher). Immunoblotting of 10 μg protein was performed under denaturing and reducing conditions using 4–12% acrylamide gels (NuPAGE™ Bis-Tris Protein Gels, ThermoFisher) and polyvinylidene fluoride membranes. Expression of LIMCH1 (NBP2–39006, Novus Biotechnology) was expressed relative to GAPDH (AF5718, R&D systems).

### 2.14 Statistical methods

In this study, experiments were performed with at least three independent biological replicates unless mentioned otherwise and the results are presented as mean ± standard deviation (SD). The normality of the data was assessed using the Shapiro-Wilk test in Graphpad Prism version 10.2.3. All datasets were found to follow a normal (Gaussian) distribution unless stated otherwise. The statistical analysis was performed with GraphPad Prism version 10.2.3 and the details regarding the statistical analysis for all data are provided in the corresponding figure legends. Differences were considered statistically significant for an at least 95% confidence level, *i.e. p* < 0.05.

## 3 Results

### 3.1 Description and performance characterization of the ALICE Cloud MAX aerosol-cell exposure system

In this study, first the equivalence of the ALICE Cloud MAX prototype and the VITROCELL® Cloud MAX with respect to aerosol (drug) delivery performance was established. Subsequently, preclinical cell-based testing of the active drug (LIMCH1-ASO) was performed with the prototype of the Cloud MAX technology developed at Helmholtz Munich, the ALICE Cloud MAX. Henceforth, the term Cloud MAX will refer to both types of Cloud MAX devices unless stated otherwise.

#### 3.1.1 Performance of the cloud MAX devices for small volume nebulizations

The design of the Cloud MAX instruments is shown in Fig. 1. The core element of the Cloud MAX technology is an exposure manifold that holds (up to) six vertically oriented cylindrical exposure tubes with a single 6-well transwell insert at the bottom. Each exposure tube can be filled with liquid aerosol from the top by connecting an airless (no airflow) vibrating mesh nebulizer *via* an adapter. Currently, any of the available Aeroneb nebulizers (Aeroneb Lab/Pro/Solo; Aerogen Inc., Galway, Ireland) can be used. Within a 1–2 min, the aerosol nebulized into each of the exposure tubes gradually settles by gravitational sedimentation to the bottom of the tube where the transwell inserts with the ALI-cultured cells are located. The sequential exposure of all six transwell inserts as described in the Methods section took 7.5 min (see Fig. 1A). Yet, if each exposure tube is operated with a dedicated nebulizer the total exposure time reduces to *ca.* 3 min (see Fig. 1B and C).

For characterization of the aerosol (drug) delivery performance of the Cloud MAX, the repeatability of aerosol-insert deposition in each of the exposure tubes was investigated by performing repeated nebulizations (*n* = 9 or 12) of 10 μL of a fluorescein solution for both the ALICE Cloud MAX and the VITROCELL ® Cloud MAX (40 mm column height). For the following sets of experiments, the transwell inserts (1 μm pore size; prevents loss of liquid through porous membrane of insert) were prefilled with 1 mL of PBS for collecting the aerosol which would have otherwise deposited onto the cells. For each nebulization run, all six inserts were sequentially exposed to aerosols, and the insert-deposited aerosol dose (volume) was measured by quantitative fluorescence spectroscopy of fluorescein and normalized to the invested solution volume (10 μL). For the ALICE Cloud MAX, the average (over all six inserts per exposure run) aerosol deposition efficiency was 38 ± 10% (mean ± 2 SD (95% confidence interval, CI); coefficient of variation CV = 13%-rel.; *n* = 12) with insert-specific mean values ranging from 29% to 45% (Fig. 2A). Similarly, the VITROCELL® Cloud MAX exhibited an average aerosol deposition efficiency of 34 ± 8% (mean ± 2 SD (95% CI), CV = 12%-rel.; n = 9) ranging between 28% and 39% (Fig. 2C). Thus, there was no statistically significant difference in performance of the Cloud MAX devices with respect to average aerosol deposition efficiency.

**Fig. 2:**
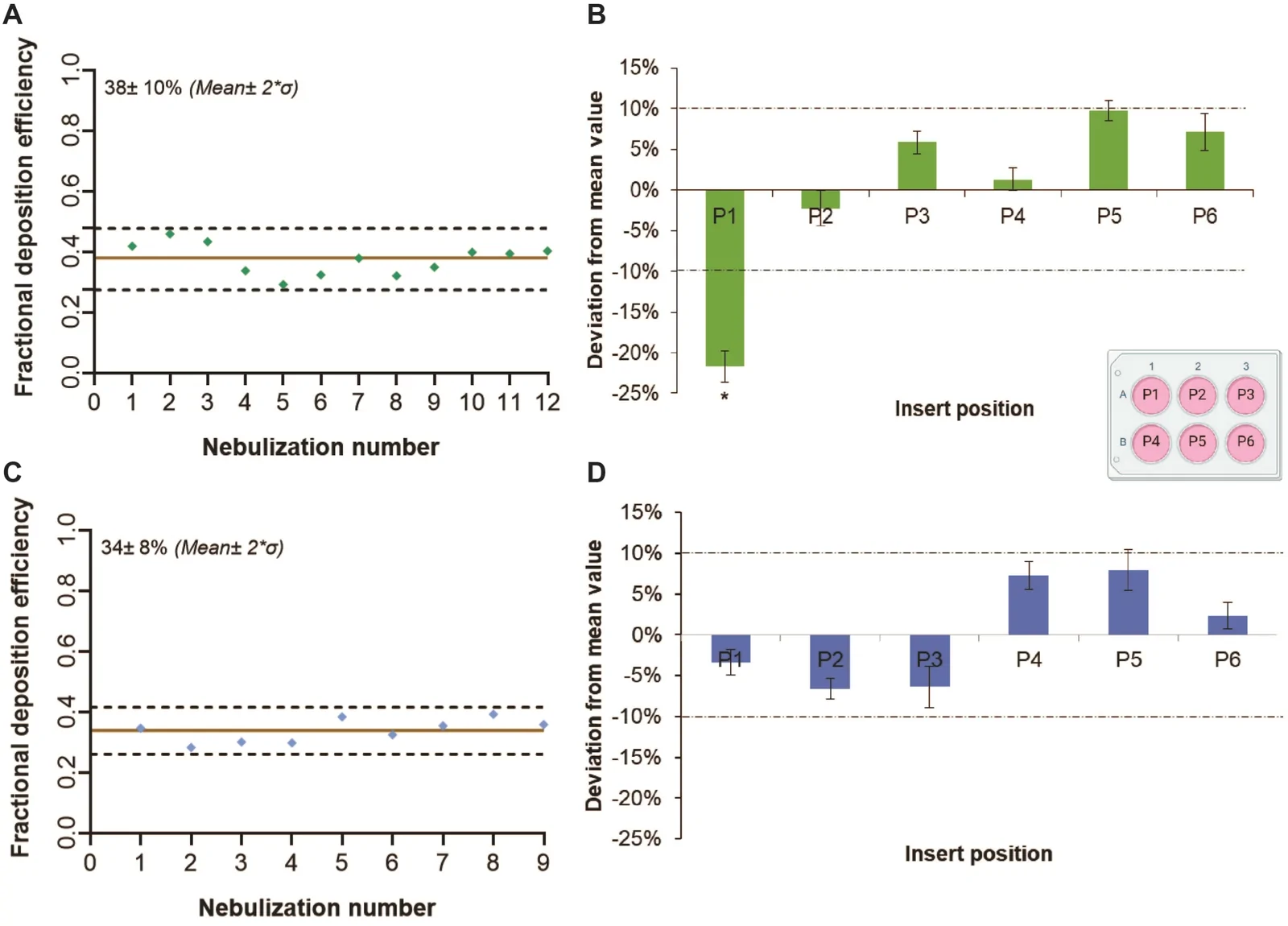
Repeatability of aerosol deposition efficiency of the ALICE Cloud MAX and the VITROCELL® Cloud MAX (40 mm tube) for nebulization of 10 μL fluorescein solution. (A) The fractional deposition efficiency averaged over all six inserts and (B) the insert-specific deviation from of the overall mean deposition efficiency for each exposure run using the ALICE Cloud MAX device. Data presented as mean ± 2 SD, *n* = 12 independent nebulizations. Panels (C) and (D) are analogous to panels (A) and (B) but for the VITROCELL® Cloud MAX. Data presented as mean ± 2 SD, *n* = 9 independent nebulizations. For panel (B) and (D), the statistical analysis was performed using one-way ANOVA with Holm- Sidak's multiple comparisons test. Inserts displaying a more than ±10% dose bias are indicated with *p-*value <0.05 (*).

Another critical performance parameter of aerosol-cell exposure systems is the insert-to-insert dose variability, which evaluates whether specific inserts receive significantly higher or lower doses than the all-insert-averaged mean dose. This was assessed by analyzing the data from Fig. 2A and C in a modified manner. Rather than averaging over all inserts for any given nebulization experiment, the relative deviations of the insert-specific mean (± 2 SD) deposition efficiency from the overall averaged deposition efficiency was calculated, and these deviations were then averaged over all exposure runs and insert positions. For the ALICE Cloud MAX, moderate insert-specific deviations in dosing of less than 10% were observed except for insert position 1 (P1) receiving 22% less than the overall mean dose (Fig. 2B). For the VITROCELL® Cloud MAX all six inserts displayed a rather modest insert-specific dose deviation ranging between −7% and 8% (Fig. 2D). Although most of the measured insert-specific dose biases were statistically significantly different from the overall mean dose (*i.e.*, different from the 0% line), dose changes of less than 10% have typically no statistically significant effect on most biological assays (*e.g.*, WST-1, LDH, IL-8 mRNA expression). This implies that only the dose-bias of the P1 insert in the ALICE Cloud MAX (−22%) is considered not acceptable (Fig. 2B). In an attempt to identify the reason for this relatively high bias in P1, everything possible was swapped including the nebulizer, the adaptor, the insert, and the order of nebulization (*i.e.*, nebulization was started with P6 instead of P1). None of these measures resolved this issue, but it will be revisited below, when the performance of the ALICE Cloud MAX is explored for another test substance, the LIMCH1-ASO.

The aerosol deposition efficiency averaged over all six transwell inserts of the ALICE Cloud MAX was assessed after nebulization of even smaller volumes of fluorescein solution (3–10 μL). With a mean deposition value of 44% (SD = 8%, coefficient of variation CV = 18%-rel.) relatively small yet statistically significant differences in mean deposition efficiency were observed depending on both nebulized volume and height of the exposure tubes (Fig. 3A). For many biological assays (including all assays used here), this relatively small dose variability of can be neglected. Yet, it is evident that nebulization of the smallest amounts of liquid, namely 3 μL (for 20 mm tube length only) and 5 μL (for 20 mm and 40 mm tube length), increases the mean deposition efficiency to 52% (SD = 4%, CV = 8%-rel.) as compared to the mean deposition efficiency of 39% (SD = 4%, CV = 10%-rel.) for 7.5 and 10 μL of liquid. Thus, nebulization of extremely small volumes of 5 μL or even 3 μL (the latter for 20 mm tube length only) maximizes the deposition efficiency (52%), but not the cell-delivered dose and dose rate, which are increasing with invested liquid volume at least up to the highest nebulized volume of 20 μL.

**Fig. 3:**
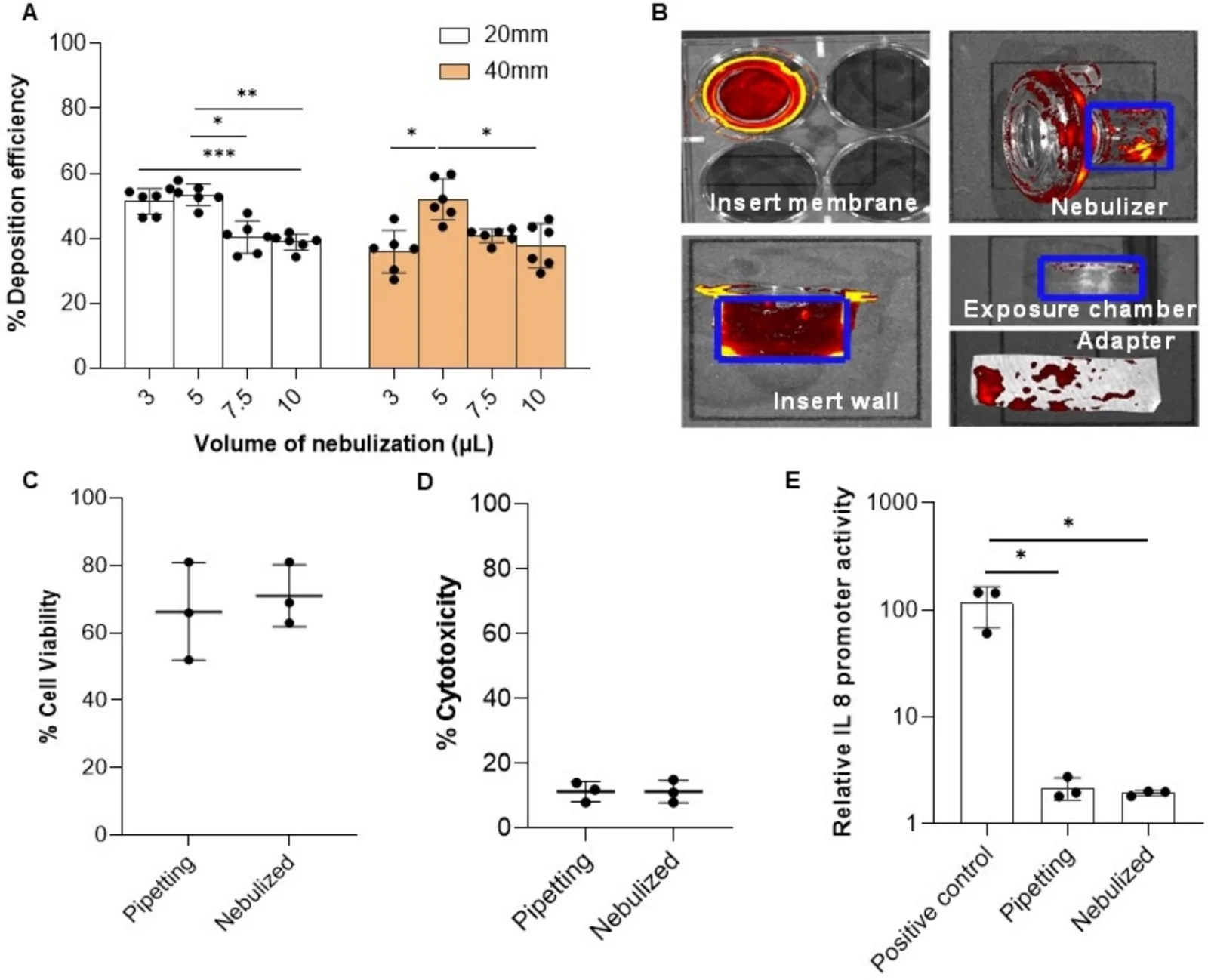
Performance of ALICE Cloud MAX device with respect to aerosol deposition efficiency, spatial uniformity of aerosol deposition within an insert, and adverse biological effects of sham nebulization on A549-Luc-IL8 cells under ALI condition. (A) Fractional aerosol deposition on the transwell insert for 3–10 μL of fluorescein solution using two different heights of the exposure tubes (20 mm and 40 mm). Statistically significant yet moderate by magnitude (mean (SD) = 44% (8%)) differences were observed. Data presented as mean ± SD (*n* = 6). (B) Representative fluorescence images depicting spatially resolved aerosol deposition on the membrane of the insert (location of cells, center region), lateral walls of the insert, nebulizer, lateral walls of the exposure tube, and adapter after nebulization of 5 μL fluorescent (SkyBlue) nanoparticle suspension with an exposure manifold of 20 mm column height. At 24 h after a sham exposure with either nebulized (20 μL) or pipetted (100 μL) of 0.9% saline of ALI-cultured A549-Luc-IL8 cells (40 mm exposure tube) there was no adverse effect on both (C) cell viability (WST-1) and (D) cytotoxicity (LDH) relative to submerged culture conditions (for 100% cytotoxicity cells were lysed). Data presented as mean ± SD (*n* = 3). (E) As a potentially more sensitive toxicity test, the mRNA expression of the pro-inflammatory cytokine interleukin 8 (IL-8) was determined 24 h post sham nebulization on A549-Luc-IL8 cells under ALI condition using a luciferase assay. As positive control, submerged-cultured A549 cells were stimulated with TNFα (15 ng/mL) for 24 h *via* the basolateral compartment of the insert. For figure panel (A) and (E), the statistical analysis was performed using one way ANOVA with Holm-Sidak's multiple comparisons test. For figure panel (C) and (D), the statistical analysis was performed using paired student *t-*test. Significance shown when *p-* value <0.05 (*), *p-* value <0.01 (**), and *p-* value <0.001 (***).

Another relevant performance parameter is the spatial distribution of the deposited aerosol over the cell-covered area of the transwell inserts. After nebulization of 5 μL SkyBlue nanoparticle suspension (fluorescein loses most of its fluorescence intensity upon drying) the fluorescence signal (dose) was found to be uniform over the cell-covered area (membrane) of the transwell inserts (see inner circular area in the top left image in Fig. 3B) (ALICE Cloud MAX - 20 mm tube). Here the shortest tube was selected, since non-homogeneous aerosol deposition due to direct inertial impact of aerosols emitted by the nebulizer is most likely for the shortest distance between nebulizer and insert. The relatively high signal from the annular region surrounding the membrane is mainly due to autofluorescence from the plastic of the lateral walls of the insert as evidenced by the rather high (yellow) signal obtained from the side-view of the insert (bottom left image in Fig. 3B). This becomes obvious when recognizing the very same “yellow” hotspot features in the non-exposed inserts (Fig. S2, top left bottom left images). Relatively low fluorescence signal (red) was also observed on the walls of the nebulizer especially in the exit tube of the nebulizer (top right image in Fig. 3B) as well as in the exposure tube/chamber and the adapter (bottom right image in Fig. 3B). Prior to nebulization almost no signal was detected on any of these surfaces except for some autofluorescence from the wall material, mainly the annular region around the membrane of the insert imaged in top view and the top part of the nebulizer (Fig. S2, top right and bottom right image). In general, aerosol deposits uniformly onto the cell covered area of the transwell insert.

For performing reliable drug testing with the Cloud MAX device, it is essential that the aerosol exposure itself does not induce any adverse effects on cells (here: A549-Luc-IL8 cells). Post sham nebulization, the cell viability (WST-1), cytotoxicity (LDH), and inflammatory response (IL-8 mRNA expression) were assessed in A549-Luc-IL8 cells cultured at the ALI condition. At 24 h after a sham nebulization with 10 μL of saline (0.9% NaCl solution), the cell viability of 66% was not statistically different from the 71% observed after pipetting 100 μL of (non-nebulized) saline on the apical side of the ALI-cultured cells (Fig. 3C). The moderately, yet statistically significant reduced values of viability near 70% are due to low A549 cell division during 48 h ALI conditions (24 h acclimatization; 24 h incubation after sham exposure) as compared to submerged incubator control conditions (100% reference) (Lenz et al., 2014). Neither nebulized nor non-nebulized sham exposure induced cytotoxicity, since the observed <15% of LDH release into the cell culture medium is below the typically accepted toxicity threshold (<20%) (Fig. 3D). Even for the typically more sensitive release of the pro-inflammatory cytokine IL-8 (Lenz et al., 2014), no statistical difference between nebulized and non-nebulized (pipetted) conditions was observed at a level which is *ca.* 100-fold lower than that of the positive control represented by TNFα (15 ng/mL) treatment for 24 h (Fig. 3E).

#### 3.1.2 Performance of ALICE Cloud MAX for aerosolized ASO delivery

In preparation of the biological LIMCH1-ASO studies with the ALICE Cloud MAX (40 mm exposure tube), the same drug delivery performance study as presented for fluorescein (Fig. 2A and C) was performed for the fluorescently labeled ASO (Cy5-ASO). For this purpose, all six inserts were repeatedly exposed to 7 μL of fluorescein or Cy5-ASO solution using the Aeroneb Solo nebulizer (20 μL was nebulized; 33% deposition efficiency - see below) as described above and in the Methods section. The fluorescein solution nebulization revealed an overall mean deposition efficiency of 36 ± 9% (mean ± 2SD, CV = 13%-rel., *n* = 10) averaged over all inserts (Fig. 4A), which is in agreement with the respective value of 39% (SD = 4%, CV = 10%-rel., *n* = 22) for nebulization of 7.5 and 10 μL of fluorescein solution (Fig. 3A) and with the 10 μL fluorescein data presented in Fig. 2A (38 ± 10% (mean ± 2 SD; CV = 13%-rel.; *n* = 12). The corresponding overall mean deposition efficiency for the Cy5-ASO solution was 33 ± 7% (mean ± 2SD, CV = 11%-rel.; n = 10) (Fig. 4C) indicating no statistical difference of the ALICE Cloud MAX performance for fluorescein and the Cy5-ASO. Analysis of potential insert-specific dose biases for Cy5-ASO according to the method described above (Fig. 2B and D) revealed that none of the inserts displayed more than 10% dose bias relative to the overall mean dose (Fig. 4C). In contrast, analogous to the 10 μL fluorescein data presented above (Fig. 2B), 20 μL fluorescein nebulization again showed more than 10% negative dose bias for the insert in position 1 only (Fig. 4B), albeit with −13% the bias is somewhat lower than the −22% observed for 10 μL fluorescein. The origin of this dose bias, which appeared for fluorescein only, remains unclear. For pharmaco−/biokinetics studies and target engagement studies with the LIMCH1-ASO no insert-specific correction for dose bias had to be applied.

**Fig. 4:**
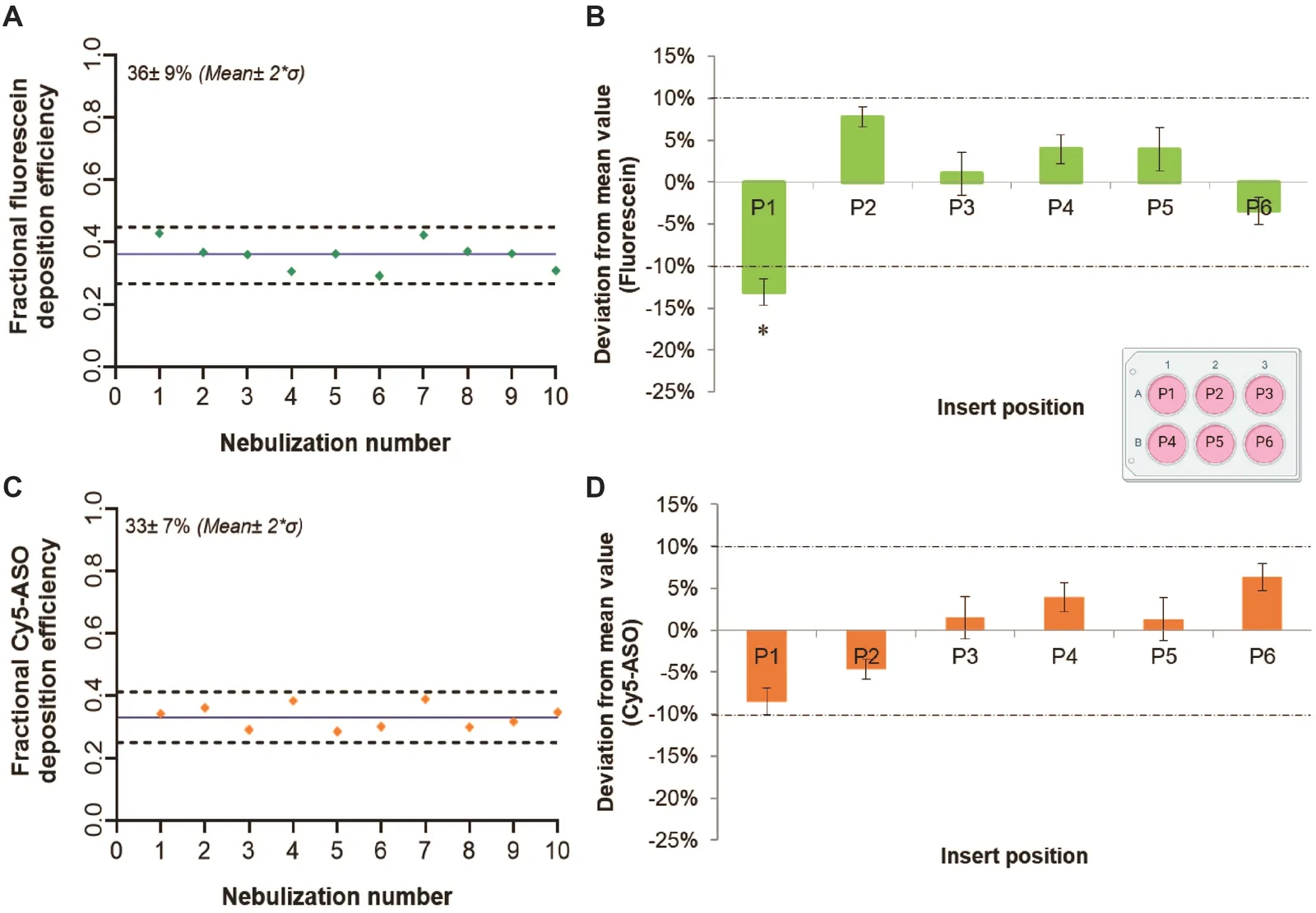
Comparative assessment of aerosol deposition on transwell inserts in the ALICE Cloud MAX device (40 mm tube) after nebulization of 20 μL of fluorescein or Cy5-ASO solution. (A) For 10 independent nebulizations of fluorescein solution, the fractional deposition efficiency averaged overall all six inserts was measured by fluorescence intensity and (B) the relative deviation of the dose in each insert with respect to the six-insert average deposition efficiency for each nebulization run was determined. Panels (C) and (D) represent the same results as seen in panels (A) and (B), but for the Cy5-ASO solution. Data presented as mean ± SD, *n* = 10 independent nebulizations. For figure panel (B) and (D), the statistical analysis was performed using one-way ANOVA with Holm- Sidak's multiple comparisons test. Inserts displaying a more than ±10% dose bias are indicated with *p* <0.05 (*).

Since all of the measured insert-averaged deposition efficiencies for 7.5 and 20 μL nebulized volume agree within statistical uncertainties, the overall insert-averaged deposition efficiency of 39% (SD = 4.2%; CV = 11%-rel., *n* = 54) was calculated from the n-weighted averages of the mean deposition efficiencies reported in Figs. 2A, C, 3A, 4A, and C). This value is independent of the type of Cloud MAX (VITROCELL or prototype), length of the exposure tube, and type of liquid (fluorescein, Cy5-ASO). Yet, a 1.3-fold larger deposition efficiency of 52% (SD = 4%, CV = 8%-rel.) was observed for 3 μL (20 mm tube length) and 5 μL (20- and 40-mm tube length) of nebulized substance.

### 3.2 *In vitro* cell-based assessment of ALICE Cloud-mediated ASO delivery and efficacy across an alveolar epithelial barrier model

#### 3.2.1 Biokinetics of LIMCH1-ASO across an alveolar epithelial barrier

Prior to biokinetics measurements, the toxicity of the ASO application was investigated. The results demonstrated that the aerosolized administration (7 μL) was non-toxic with respect to both cell viability (WST-1 assay) and cytotoxicity (LDH assay) for both the mono- and the co-culture cell model (Fig. S3). For the non-physiological but frequently employed pipetting application (100 μL), no toxic effect was observed in the monoculture model (Figs. S3A, and B). In contrast, in the co-culture model, mild toxicity was detected under both pipetting and nebulization conditions, with cytotoxicity levels of approximately 20–25%, accompanied by elevated metabolic activity (WST-1 values ranging from 130% to 200%) as often seed for mild levels of cytotoxicity which can be counteracted by enhanced metabolic activity of the cells (Figs. S3C, and D). Overall, these findings indicate that LIMCH1 downregulation does not adversely affect cell viability or induce cytotoxicity.

To assess the engagement of the LIMCH1-ASO target in an IPF patient, the conditions of inhalation therapy were mimicked by nebulizing LIMCH1-ASO onto an *in vitro* co-culture model consisting of alveolar epithelial cells (A549), which shield IPF-derived fibroblasts from direct contact with the aerosol-delivered drug. Therefore, the LIMCH1-ASO must cross the A549 cell barrier to reach the IPF effector cell (fibroblasts). Fig. 5A shows the workflow for establishing the co-culture model, and Fig. 5B depicts the confluence of the apical A549 barrier and the elongated shape of the fibroblasts on the basal side as evident from immunohistochemical microscopy. To measure the longitudinal transport of the fluorescently labeled Cy5-ASO through the ALI-cultured A549 monolayer to the fibroblasts, a non-toxic dose of 3 nmol of Cy5-ASO was delivered to the apical side of A549 monoculture (no fibroblasts). The corresponding gradual increase of Cy5-ASO in the basal compartment (normalized to the delivered Cy5-ASO dose) was monitored by spectrofluorometer between 15 min and 72 h (0.25–72 h) (Fig. 5C, D). In this experiment, either 20 μL of fluorescently labelled ASO (Cy5-ASO) was nebulized using the ALICE Cloud MAX (7 μL depositing on the cells) or 100 μL of Cy5-ASO solution was pipetted onto the apical side of the ALI-cultured A549 cells. The latter condition was included to assess the influence of the applied liquid volume on the observed biokinetic behaviour.

**Fig. 5:**
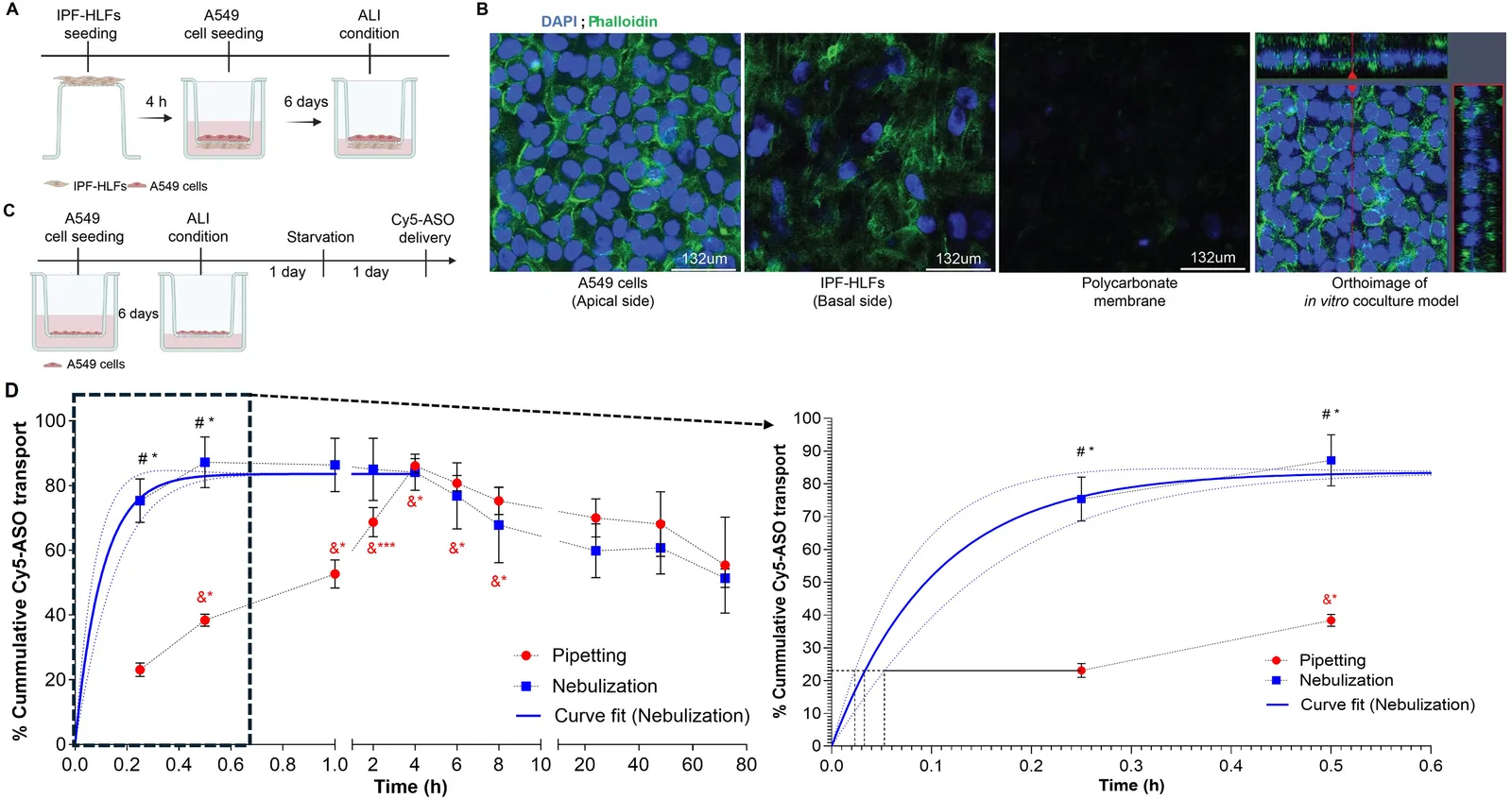
An *in vitro* co-culture model mimicking the alveolar tissue consisting of primary human fibroblasts from IPF patients (IPF-HLFs) covered with an alveolar epithelial cell barrier (A549) under ALI condition was used for studies on LIMCH1-ASO target engagement and biokinetics studies (without fibroblasts). (A) A schematic overview of establishing the alveolar co-culture *in vitro* model under ALI culture conditions consisting of A549 and IPF-HLF cells. (B) Representative immunofluorescent staining of cell nuclei (DAPI, blue) and F- actin cytoskeleton (Phalloidin, green) on both sides of the co-culture model (apical - A549; basal - fibroblasts) just prior to Cy5-ASO delivery (see Fig. 5C). Scale bar: 132 μm. For biokinetics studies, the A549 monoculture was cultured as depicted in (C) and (D) shows the observed biokinetics profile of fluorescent Cy5-ASO covered with a confluent A549 monolayer, which represents the epithelial barrier that ASOs must cross to reach the fibroblasts (not seeded here) in the co-culture model. Measurements of the cumulative Cy5-ASO dose in the basal compartment were performed for up to 72 h after the application of Cy5-ASO either *via* nebulization (7 μL) or pipetting (100 μL). To estimate the time for transport of 23% Cy5-ASO after nebulization (*t*_*23*_), the corresponding time point was extrapolated from an exponential curve fit the data up to 6 h (solid line – see zoomed-in figure on the right), resulting in an estimated transport time of 0.034 h (121 s; 95% CI: 0.015 to 0.046 h). Data are presented as mean ± SD, *n* = 3. Statistical analysis was performed using one-way ANOVA with Holm-Sidak's multiple comparisons test. Significance shown when *p-* value <0.05 (*), *p-* value <0.01 (**), and *p-* value <0.001 (***). #: time-matched comparison between nebulization *vs.* pipetting ASO application; &: comparison between all timepoints *vs.* 0.25 h time-point within each ASO application route. (For interpretation of the references to colour in this figure legend, the reader is referred to the web version of this article.)

As shown in Fig. 5D, the maximum basal dose was between 83% and 86% of the Cy5-ASO dose deposited onto the cells for both routes of delivery. For nebulization delivery, the peak value was reached within 15 min (0.25 h), since this value was not significantly different from the “peak” value observed at 1 h. In contrast, for pipetted delivery the maximum basal dose was reached between 2 and 4 h, *i.e.*, nebulization exhibited a much faster transepithelial transport kinetics of Cy5-ASO than the pipetting approach. The observed rapid and high cumulative transport of the aerosolized Cy5-ASO across the epithelial barrier suggests that the A549 barrier was relatively “leaky” with respect to the relatively large Cy5-ASO molecules (6151 Da) with a van-der-Waals volume diameter of 5.2 nm. Interestingly, the Cy5-ASO dose observed at the basal compartment decreased after 4 h in a gradual monotonic way until 72 h for both types of ASO application (Fig. 5D). As this decline is not dependent on the type of ASO application (see 4 h to 72 h period), it is attributed to wall losses of the Cy5-ASO in the basal compartment.

At 4 h, small amounts of ASO were still detected in both the apical (5.2% for the pipetting application and 6.4% for nebulization) and the cell compartment (1.3% for pipetting and 2.1% for nebulization) yielding a total non-basal delivered dose of 6.4% and 8.5% for pipetting and nebulized application, respectively. Combined with up to 84–86% of ASO detected in the basal compartment and an estimated wall loss of less than 14% in the basal compartment (based on the observed decline in the basal dose between 4 h and 8 h). This suggests that, within experimental uncertainties, 100% of the delivered ASO dose could be accounted for in the basal, apical and cell compartment. Thus, between 91.5% and 95.6% of the delivered dose penetrated the A549 epithelial barrier within 15 min (0.25 h) for nebulized application and between 2 and 4 h for pipetted application. This also implies that there was zero ASO attachment to the porous membrane of the insert.

#### 3.2.2 ASO mediated *LIMCH1* knockdown

Target engagement was assessed in both the mono- and co-culture model of primary IPF fibroblasts. In the monoculture model, no significant differences were observed between pipetted and nebulized ASO delivery with respect to *LIMCH1* transcript downregulation. Consequently, data from both ASO delivery approaches were pooled for subsequent analysis. For the 3 nmol dose, the non-targeting control oligonucleotide exhibited an overall mean expression of 0.96 ± 0.16 (mean ± SD), whereas the LIMCH1-ASO condition showed a markedly reduced mean expression of 0.081 ± 0.030, corresponding to a 12-fold downregulation. Notably, at the 15 nmol LIMCH1-ASO dose, the *LIMCH1* transcript levels were downregulated below the detection limit (negative values) for both conditions (Fig. 6B). Conversely, in the coculture model, generally less *LIMCH1* silencing was observed and especially for 3 nmol pipetted ASO delivery was less efficient than nebulized delivery. At the 3 nmol dose, the non targeting control oligonucleotide showed an overall mean expression of 0.96 ± 0.10, whereas there was reduced overall men expression of 0.50 ± 0.097, corresponding to a 2-fold downregulation for pipetted application and for nebulized delivery the same downregulation as for monocultures (below detection limit) was detected. At the 15 nmol dose, the non targeting control oligonucleotide displayed a mean expression of 1.04 ± 0.11, while the LIMCH1-ASO condition was reduced to 0.23 ± 0.042 (pooled nebulized and pipetted delivery), corresponding to a 4.5-fold downregulation irrespective of delivery route (Fig. 6C). This 3- to 6-fold reduced *LIMCH1* downregulation due to the presence of A549 cells (covering the fibroblasts) indicates a significant efficacy shielding effect for both 3 nmol and 15 nmol. The efficacy of the ASO was further validated at the protein level in the coculture *in vitro* model. Due to low statistical power (*n* = 2 for each setting), the data from the two delivery routes had to be pooled for analysis. At 3 nmol dose, the non targeting control oligonucleotide revealed an overall mean expression of 0.98 ± 0.33, which was reduced significantly to 0.20 ± 0.081 upon LIMCH1-ASO treatment, corresponding to a 5-fold reduction. At the 15 nmol dose, the non-targeting control oligonucleotide displayed a mean expression of 1.11 ± 0.080 and was reduced to 0.06 ± 0.028 upon LIMCH1-ASO treatment, corresponding to a 18.5-fold reduction (Fig. 6D, E). Taken together, the LIMCH1-ASO demonstrated target engagement and downregulation of LIMCH1 protein levels in both IPF fibroblast cell models, which was independent of the route of delivery (aerosol: 7 μL; pipetting: 100 μL) for non-A549 protected fibroblasts. However, for the co-culture model, a 3- to 6-fold reduced LIMCH1 mRNA downregulation (as compared to the fibroblast monoculture) was observed and for 3 nmol the nebulization of ASO was more efficient than pipetting. Enhanced effects on the mRNA levels in the monoculture model can be interpreted as “shielding” of the fibroblasts from drug exposure due to the presence of A549 alveolar epithelial cells.

**Fig. 6:**
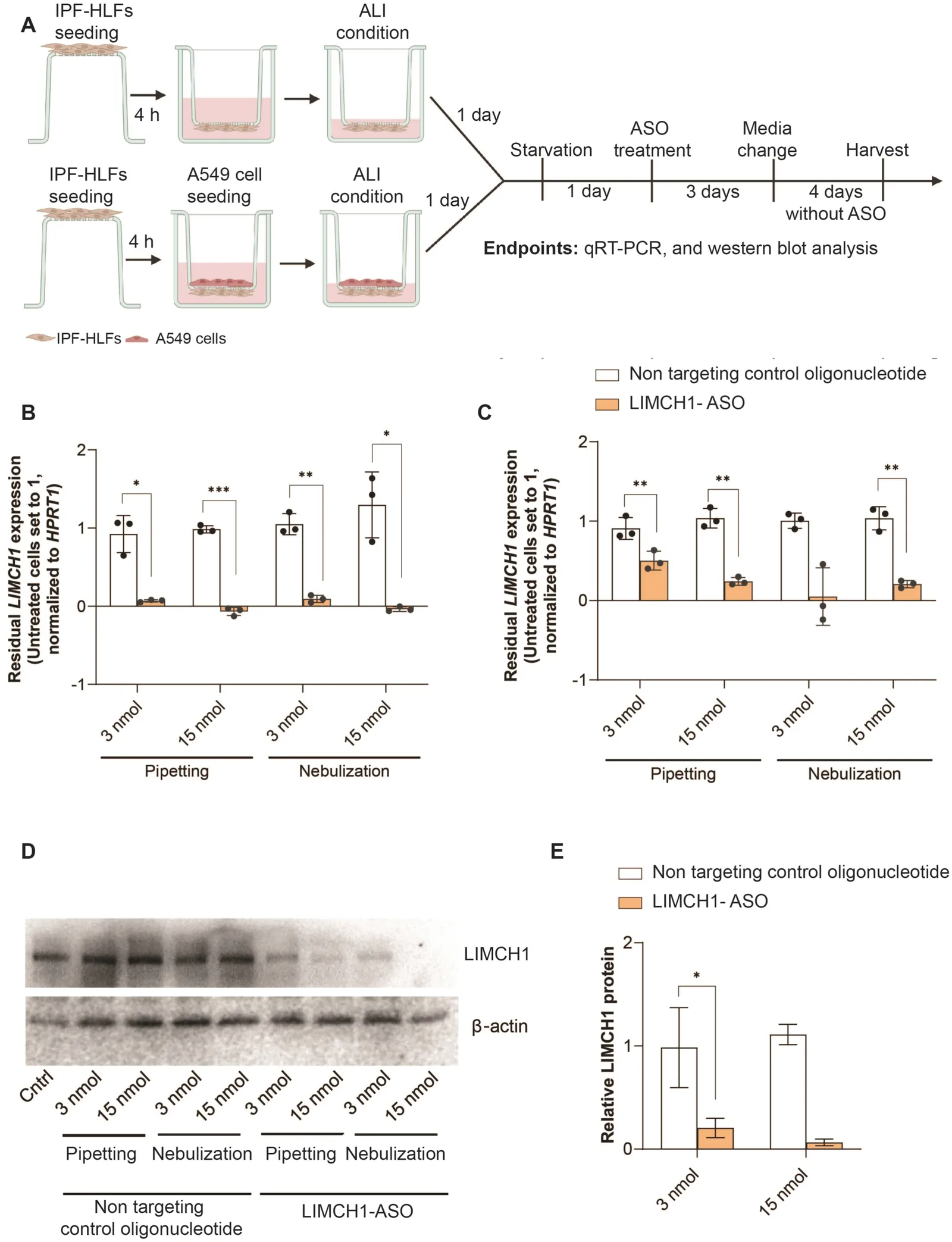
LIMCH1-ASO treatment reduces LIMCH1 expression at the transcript and protein levels in both mono- and co-cultures of IPF-derived human lung fibroblasts. (A) The experimental approach for the transcript analysis of the human *LIMCH1* using bDNA assay in both (B) monoculture *in vitro* model of IPF-HLFs and (C) co-culture *in vitro* model consisting of A549 and IPF HLF cells, following the application of LIMCH1-ASO at two different doses of 3 nmol (*i.e.*, 0.64 nmol/cm^2^) or 15 nmol (*i.e.*, 3.21 nmol/cm^2^) either *via* nebulization (7 μL) or pipetting (100 μL). Data are presented as mean ± SD, *n* = 3. An outlier test was performed on the technical replicates of a single biological replicate for LIMCH1 bDNA data for 3 nmol LIMCH1-ASO nebulization condition in the monoculture *in vitro* model using GraphPad Prism. (D) Western blot analysis and (E) related quantification of the human LIMCH1 protein levels in the co-culture *in vitro* model observed in primary IPF-HLFs, following LIMCH1-ASO application at two different doses of 3 nmol (*i.e.*, 0.64 nmol/cm^2^) or 15 nmol (*i.e.*, 3.21 nmol/cm^2^) either *via* nebulization (7 μL) or pipetting (100 μL). Data are presented as mean ± SD, *n* = 2. For panel (B) and (C), the statistical analysis was performed using paired student *t-*test. For (E), the western blot quantification data from both pipetting and nebulization conditions were combined for the 3 nmol and 15 nmol ASO applications, and the statistical analysis was performed using a paired student *t-*test. The combined dataset for 15 nmol LIMCH1-ASO did not follow normal gaussian distribution; therefore, a nonparametric Wilcoxon test was used, revealing no significant difference compared with the 15 nmol non-targeting control oligonucleotide. Differences between LIMCH1-ASO delivery *via* pipetting and nebulization for the above-mentioned ASO doses are presented in the Supplementary Fig. S5. Significance levels: *p* <0.05 (*), *p* <0.01 (**), and *p* <0.001 (***).

To further confirm the ability of ASOs to downregulate *LIMCH1* in lung fibroblasts, a second type of human lung fibroblasts (CCD-16Lu cells) was used and explored the relevance of ASO application (either *via* pipetting or nebulization). First the non-toxic dose level of LIMCH1-ASO was found to be 1 μM (Dose: 0.29 nmol/cm^2^) for CCD-16Lu cells with respect to cell viability (Fig. S4A). Application of non-toxic LIMCH1-ASO exposure levels resulted in a 9.5-fold downregulation of *LIMCH1* mRNA relative to the non-targeting control oligonucleotide (Fig. S4B). At the protein level, the downregulation was attenuated, with a 5-fold reduction compared to the non-targeting control oligonucleotide (Figs. S4C, and D). Notably, pipetting-mediated ASO delivery induced a more pronounced *LIMCH1* downregulation (7-fold), whereas nebulization resulted in a 4-fold downregulation (Fig. S4E). Taken together, these findings suggest that low volume ASO application effectively downregulates *LIMCH1* in human lung fibroblasts *in vitro,* regardless of the route of administration.

## 4 Discussion

The main driver of the currently observed intense research activities towards more advanced and complex *in vitro* models of the lung is due to the demand of increasing more physiological and clinically relevant models with an expected higher propensity for clinical validation. One of the main objectives of these activities is the reduction or even replacement of animal testing in the preclinical drug development process. Often new technological developments are bioinspired, thus as the use of air-liquid interface over submerged culture conditions for lung epithelial cells, 3D arrangement of different types of cells representing different 3D organ niches (*e.g.* the alveolar air-blood barrier can be mimicked by stratified arrangement of macrophages, epithelium, fibroblasts, endothelium) or expression of cell-specific *in vivo* molecular marker profiles (Ehrmann et al., 2020; Darquenne et al., 2016; Hajari et al., 2025). Yet, the demonstration of improved clinical relevance on a functional level is often difficult to provide. Following physiological similarity considerations, it is evident that aerosolized drug delivery directly onto ALI-cultured lung epithelial cells with the Cloud MAX technology is more accurately biomimicking the conditions in the lung during inhalation therapy. In this section the utility and clinical relevance of the Cloud MAX technology is discussed with particular focus on the repeatability of dosing and small-volume nebulized drug delivery. Functional relevance will be demonstrated by revealing the reasons for the previously poorly understood “burst-like” pharmaco−/biokinetics profiles of nebulized drugs and the possibility of epithelial barriers “shielding” fibroblasts from inhaled drugs like LIMCH1-ASOs potentially resulting in reduced drug efficacy as compared to exposure of fibroblasts alone.

### 4.1 Cloud MAX aerosol cell-exposure system

Cloud-based aerosol-cell exposure (or administration) systems have a proven track record of successful *in vitro* testing of inhalable substance under clinically relevant conditions with aerosolized substance delivery to ALI-cultured cells. For example, ALICE/VITROCELL® Cloud technology has been used for preclinical testing of various substances related to nanoparticle toxicity (Bannuscher et al., 2022; Stoleriu et al., 2024; Chen et al., 2024; Barosova et al., 2020; Braakhuis et al., 2023; Hufnagel et al., 2020; Leroux et al., 2022; Mastalerz et al., 2022) and to a lesser extent for testing drugs focusing on small molecules, such as bortezomib, nintedanib, 2-deoxylated glucose analogues, and liposomal ciclosporin (Doryab et al., 2022; Lenz et al., 2014; Leroux et al., 2022; Schmid et al., 2017; Wideman et al., 2025), as well as a few studies with biologics such as IgG (Röhm et al., 2017; Rittersberger et al., 2025). The relatively small number of studies with “expensive” drugs and biologics is likely due, at least in part, to the inefficient use of substances in most aerosol-cell delivery systems compared to the pipetting drugs into the medium of submerged cell culture models, which yields a nominal efficiency of 100%. However, the effective drug delivery efficiency may be much lower than 100% especially for hydrophobic (amphiphilic) or nanoparticle-packaged drugs which may reside in the medium without contacting cells (Teeguarden et al., 2007). Cloud based aerosol-cell administration systems excel with respect to drug delivery efficiency and rate. Technologies that use cloud settling as the main aerosol-to-cell deposition mechanism have been shown to deliver doses up to 100 times faster and more efficiently than devices that rely on single-particle deposition due to gravitational settling or even electrostatically or thermophoretically enhanced deposition (Paur et al., 2011; Ritter et al., 2023). This is because a dense cloud of aerosol has a much higher settling velocity than a single particle of the same size due to reduced drag forces acting on aerosols inside the cloud (wind shield effect) (Lenz et al., 2009). Most currently available cloud-based systems require the nebulization of liquid volumes of at least 20 μL, and often more than 200 μL for reliable operation (Lenz et al., 2009; Lenz et al., 2014; Röhm et al., 2017; Horstmann et al., 2021). The Cloud MAX technology which is introduced and performance-characterized here extends the required invested volume range down to 3 μL while delivering clinically relevant drug doses to cells.

The comprehensive performance characterization of the two types of Cloud MAX systems used here (VITROCELL® Cloud MAX and its prototype ALICE Cloud MAX), revealed no statistically significant difference, which was expected, given their identical design parameters. For comparison with other aerosol-cell exposure systems, the aerosol delivery efficiency was presented as cell-deposited liquid volume normalized to the invested (nebulized) volume. According to our experience, the reported results are largely independent of drug concentrations up to about 50 mg/mL. For larger concentrations the viscosity and/or surface tension of the liquid drug may be altered with potential effects on the performance of the Aeroneb Solo nebulizer and thus of the Cloud MAX. Albeit we have demonstrated that the deposition efficiency depends on the nebulized volume it is instructive to report the overall average deposition efficiency as 44% (SD = 8%, CV = 18%-rel.) as a mean value as general orientation but with artificially enhanced CV, for more precise performance assessment the nebulized volume should be accounted for. We recommend using the more precise volume-stratified values of 39% (SD = 4.3%, CV = 11%-rel.) for nebulization of 7.5 to 20 μL and 52% (SD = 4.2%, CV = 8%-rel.) for 3 to 5 μL of nebulized liquid (excluding the 3 μL value for 40 mm tube length due to evaporation effects as indicated below). These values represent averages of the mean deposition efficiency values (and CV) measured for any given setting defined by the nebulized volume, type of nebulized liquid, as well as type and geometry (tube length 20 or 40 mm) of the Cloud MAX systems as given in Figs. 2A, C, 3A, 4A, and C. These dose deviations of CV = 11%-rel. were considered acceptable, as they did not significantly affect the biological assays reported in this study. On a general level, the biological relevance of dose deviations on *in vitro* assays depends on several factors, including compound potency in eliciting the desired biological response and the characteristics of the dose-response relationship, as outlined in the International Council for Harmonization guideline for analytical procedures (ICH Guideline Q2 (R2), 2026). If biological data obtained from different insert positions are compared (instead of averages over all inserts), the relatively small insert-specific systematic dose biases of less than 10% should be taken into account (Fig. 2B, and D). It is noteworthy that the insert at position 1 of the prototype device exhibited a notable negative bias of −22% for unknown reasons (Fig. 2B, and D). This insert position was not used for LIMCH1-ASO testing. It is important to note that this feature was not present for the commercially available VITROCELL® Cloud MAX device, which exhibited insert-specific dose bias below 10% for any of the inserts (for a 40 mm tube height).

The observed dependence of the deposition efficiency on the nebulized liquid volume can easily be taken into account using the values reported above. Yet, there is one notable “outlier”. Unlike all other nebulized volumes, for 3 μL only there is a dependence of deposition efficiency on exposure tube length. For this case the mean deposition efficiency is 52% for the short (20 mm) and 36% for the long (40 mm) tube length (Fig. 3A) which is attributed due to partial droplet evaporation. If aqueous aerosol is sprayed into a volume contain dry air (*i.e.* with relative humidity RH < 100%), these droplets will partially evaporate and thus shrink until RH reaches 100%. In the worst case, a large fraction or even all of the aerosol droplets will evaporate and may shrink to a level which reduces aerosol settling, enhances diffusive aerosol loss to the lateral walls, and thus reduces aerosol deposition efficiency on the cells. For any given liquid volume, the drying effect will be more severe in larger volumes. Thus, depending on exposure tube length and RH in the tubes there is a lower limit of nebulized liquid volume for maximal deposition efficiency. For nebulization of 3 μL this limit was reached for the larger, but not for the shorter exposure tube of the Cloud MAX. It is important to note, that this effect does not prevent using the Cloud MAX for less than 3 μL volumes as long as the deposition efficiency is reproducible. This evaporation effect will just enhance the lowering of liquid volume (dose) delivered to the cells with reduced nebulized volume yielding volume per cell area levels the 0.25 μL/cm^2^ reached for 3 μL (40 mm exposure tube) in this present study.

There are a number of other methodologies for liquid aerosol-cell delivery to ALI-cultured cells. These include continuous flow aerosol delivery technologies using diffusion, gravitational settling, and inertial impaction as deposition mechanisms, sometimes combined with electrophoretic or thermophoretic deposition. Also, air flow-free spray deposition methodologies have been described (Lacroix et al., 2018). Yet, the principle of air flow-free delivery of a well-mixed, very dense aerosol cloud containing at least 0.1 μL/cm^3^ air (condition for cloud instead of single aerosol settling) has been proven ideal for spatially uniform, fast, and dose-controlled delivery of clinically relevant (high enough) doses using small amounts of substance (Lenz et al., 2009; Lenz et al., 2014). Therefore, the following discussion focuses on devices meeting these requirements. Our lab previously reported a 51.5 ± 1.0% deposition efficiency for 10 μL air flow-free nebulization into the MALI bioreactor system (later modified and named CIVIC), which is geometrically very similar with respect to the aerosol exposure tube but suitable for mechanically stretched *in vitro* cell culture models of the lung (Doryab et al., 2022; Cei et al., 2021). This is in excellent agreement with the 52% ± 4.0% value of the Cloud MAX for 3–5 μL nebulization, but it is larger than the 39% ± 4.2% for 7.5–20 μL nebulization. Thus, the different geometry seems to introduce a change in the critical volume defining the transition for more efficient to less efficient aerosol deposition for switching from low to high volume nebulization, which is between 5 and 7.5 μL for the Cloud MAX, but at least 10 μL in the MALI system. For another air flow-free, Cloud-based device with the same type of nebulizer (Aeroneb vibrating mesh nebulizer) and a tapered cylindrical exposure chamber, a *ca.* 10-fold lower deposition efficiency of 3.2–5.5% for 12-well transwell inserts was reported, depending on the amount of nebulizer liquid (20–200 μL) (Horstmann et al., 2021). For larger rectangular exposure chambers suitable for exposing up to 2 multi-well plates with each nebulization, deposition efficiencies of up to 16% have been reported (Lenz et al., 2009; Lenz et al., 2014; Röhm et al., 2017). This indicates that currently *ca.* 55% of substance efficiency is possible but optimized geometry is important for achieving the highest delivery efficiency especially for lowest-volume nebulization.

This relatively high substance delivery efficiency is one of the main advantages of the Cloud MAX, but it also has other advantages over alternative aerosol-cell exposure systems for *in vitro* testing of often expensive experimental drugs. To the best of our knowledge, the VITROCELL® Cloud technology is the most efficient aerosol-cell delivery system (16%) which is currently available. It requires nebulization of 200 μL of liquid for simultaneous exposure of up to six 6-well inserts yielding a surface-specific dose of 1.2 μL/cm^2^ (5.4 μL/4.5 cm^2^) (Lenz et al., 2014). This same dose can be achieved with 15 μL in the Cloud MAX. For three to six exposed inserts, this corresponds to a substance use of 45–90 μL (3 × 15 μL to 6 × 15 μL) in the Cloud MAX. Thus, the Cloud MAX is 2.2- to 4.4- fold more substance-efficient than the VITROCELL® Cloud. Also, its relatively simple handling protocol ensures that technical personnel can be trained to operate the Cloud MAX system within a few hours, or its handling can be learnt directly from the procedure described in the present study.

Another clinically important aspect for physiologic drug testing is the amount of deposited liquid per area of lung epithelium during inhalation therapy. In clinical settings this value is typically below 0.1 μL/cm^2^ as rationalized in Section 4.3 below. As indicated above the currently reached lowest value with the Cloud MAX for nebulization of 3 μL (40 mm tube height) is 0.25 μL/cm^2^. It is very likely that this value can be decreased by nebulization of less than 3 μL, but this was not explored here. However, in this study and in other studies no adverse biological effects of vehicle control exposures have been reported for up to at least 4 μL/cm^2^ (Lenz et al., 2014; Röhm et al., 2017; Rittersberger et al., 2025).

The Cloud MAX technology also has a few limitations that should be considered. To ensure the highest possible throughput, short exposure times are essential. For the Cloud MAX experiments performed here, only one nebulizer was used requiring sequential nebulization into the exposure tubes, which resulted in a total exposure time of 7.5 min for six inserts. This is about 2- fold larger than the exposure time of multi-well exposure systems like the VITROCELL Cloud (alpha) technology (Lenz et al., 2014; Röhm et al., 2017). This limitation can be overcome by using six nebulizers in parallel (one for each exposure tube), which is expected to reduce the exposure time to *ca.* 3 min (2 min exposure plus positioning (un-) loading of the six inserts) (Lee et al., 2023; Wideman et al., 2025). As a caveat, it is also important to note that nebulizing more than 20 μL at a time in the Cloud MAX is not recommended, since larger volumes will eventually lead to excessive liquid deposition on all wetted walls. This may result in liquid dripping from the walls into the insert, which could compromise spatially uniform drug deposition and obscure the cell-delivered dose. However, repeated exposure to 20 μL of liquid in 2 min time intervals is possible if care is taken that the inner walls remain sufficiently dry. It is also noteworthy that the VITROCELL Cloud MAX can be adapted to hold not only one 6-well per exposure tube but also one 12- or 24-transwell inserts, yielding the same surface-specific dose (μL/cm^2^) as with 6-well inserts. However, the substance efficiency scales with the size of the cell-covered area. Since the cell-covered area reduces by factor of *ca.* 4 each when switching from 6- to 12-well inserts and from 12- to 24-well inserts, the substance efficiency also reduces by this factor.

For potential users of the Cloud MAX technology, and to facilitate its adaptation and acceptance by regulatory bodies as a new approach methodology (NAM) for the reduction and potential replacement of animal testing, we provide a concise summary of recommendations for handling the Cloud MAX system as well as an overview of the advantages and limitations of the Cloud MAX.

Recommendations for best-practice handling of Cloud MAX:

1. Nebulize liquid volumes of 3–20 μL.
2. Selection of most appropriate type of nebulizer. Depending on the physicochemical properties of the substance (viscosity, surface tension) and the type of drug (see “limitations” below for more details).
3. Performance monitoring of vibrating mesh nebulizers:
  a. Monitor nebulizer output rate (mL/min) with saline and drug formulation, since this may affect Cloud MAX performance.
  b. Replacement nebulizer within a study should have similar output rate.
  c. Change in output rate of a nebulizer may indicate cracks in the device, (partial) clogging of the vibrating mesh, failure of electronics.
4. Verify stability of solution or suspension during time period between preparation and use of nebulized substance.
5. Verify resilience of drug formulation to nebulization process. For instance, perform pipetted efficacy or toxicity assay with drug formulation prior and after nebulization as described in this study (collect nebulized drug by nebulizing directly into a 50 mL falcon tube).
6. Avoid false exposure route. Inadvertent exposure of the cells from the basolateral compartment by direct exposure of the basal cell culture medium (bypassing the cells) through open lateral walls of the insert. Solution: Use media covers for full isolation of the apical from the basal compartment or slightly lower the basolateral medium level to avoid contact between medium and cells during the short exposure period.
7. Dosimetry: Albeit the Cloud MAX has demonstrated high stability with respect to dosing, it is advisable to include appropriate dosimetry tools whenever possible especially for investigation of dose-response relationship, comparison of results with *in vivo* animal or clinical human data. (for details see “advantages” item 10).

Advantages of Cloud MAX:

1. Small volume nebulization (3–20 μL, possibly also <3 μL).
2. High substance efficiency, low cost for substance use.
3. High enough doses for therapeutic efficacy testing.
4. Short exposure times due to high dose delivery rate (*ca.* 1 μL cm^−2^ min^−1^).
5. High repeatability of dosing (CV = 8–11%.rel).
6. Readily implemented by users with no prior aerosol experience (simple workflow).
7. Simple cleaning workflow, autoclavable.
8. Suitable for broad range of transwell insert types and formats (6- to 24- well).
9. Suitable for nanocarriers and commonly used aqueous formulations and organic solvents (including DMSO).
10. Compatible with real-time and off-line dosimetry methods (*e.g.* quartz crystal microbalance, TEM grids, mass spectrometry).

Limitations of Cloud MAX:

1. Slow to medium throughput sampling: from *ca.* 1.5 min to 0.5 min/insert depending on use of one to six nebulizers.
2. Not suitable for dry powder formulations.
3. Operational limits of vibrating mesh nebulizer technology: high viscosity liquid (*ca.* 50 mg/mL or higher concentrations), degradation of structurally delicate substances (*e.g.* peptides, proteins, extracellular vesicles, lipid nanoparticles, bacteria, virus). For such cases, optimized mesh nebulizers may be required (*e.g.* lager mesh pore size).

### 4.2 Assessment of ASO biokinetics to fibroblasts across an alveolar epithelial barrier model

Currently, most *in vitro* drug efficacy studies of inhaled drugs use non-physiological submerged cell culture models. In these models, the drug is typically pipetted with 200–300 μL of medium per 1 cm^2^ of cells, creating a 2–3 mm (2000–3000 μm) thick liquid layer on the cells. This layer may bias the clinical relevance of these studies where cells are covered only by a *ca.* 80 nm layer of alveolar surfactant with an added *ca.* 40 nm layer of inhaled liquid drug (assuming 2.5 mL of an inhaled drug deposits with a 40% efficiency onto the lung epithelium (100 m^2^). For more details on this please refer to Section 4.3. To investigate the effect of dispersing ASO in a larger volume of liquid, as is encountered during submerged cell culture experiments, a pipetting application scenario was included here. In this scenario, 100 μL of ASO suspension was pipetted into a 6-well insert, yielding a volume dose of 21 μL/cm^2^ corresponding to a 210 μm thick liquid layer. This layer is 14- fold thicker than in the Cloud MAX nebulization scenario (1.5 μL/cm^2^ or a 15 μm thick layer), but it is still 10- to 15- fold thinner than in typical submerged cell culture conditions. No obstacles were encountered when using the Cloud MAX to deliver aerosolized ASOs to our ALI cell culture models, but care was taken that cells had no contact to the basal medium during the ASO aerosol exposure to avoid inadvertent transport of ASO aerosol directly into the basal medium during exposure. The output rate of the Aeroneb Solo nebulizers and the Cloud MAX's drug delivery performance were not altered by LIMCH1-ASO compared to saline/PBS (Fig. 4). No adverse effects of the Cloud MAX (saline) exposure procedure were detected for A549 cells (Figs. 3C, and D), which is a prerequisite for reliable preclinical drug development.

Very often *in vitro* comparison experiments are performed using the same concentration of drug. In a previous study with Bortezomib (proteasome inhibitor) and A549 cells under ALI and submerged culture conditions, we have shown that the IC_50_ value of the concentration-efficacy curves for aerosolized-ALI vs. pipetting-submerged delivery differed by up to 1000-fold. Yet, the IC_50_ values of the dose-efficacy curves were independent of the application route, *i.e.*, for 2D A549 cell culture models the drug dose is often a better predictor of response than concentration (Doryab et al., 2023; Lenz et al., 2014). Thus, we applied identical doses of 3 nmol and 15 nmol for the ASO efficacy and toxicity study, and 3 nmol for the biokinetics study for aerosol and pipetted drug application. To evaluate potential drug dose "shielding" effects in fibroblasts resulting from the presence of alveolar epithelial cells, the ASO biokinetics was investigated across a confluent monolayer of A549 alveolar epithelial cells. It is acknowledged that the applicability of A549 cells in pharmacokinetic studies can be debated due to their relatively low transepithelial electrical resistance (TEER; approximately 50–200 Ω cm^2^; here 130 Ω cm^2^), which is indicative of limited tight junction formation (Doryab and Schmid, 2022). Nonetheless, A549 cells have proven suitable for transepithelial transport studies involving large molecules such as Cy5-ASOs (∼6.1 kDa), where transport is primarily influenced by cell-cell adhesion rather than tight junction integrity (Lieber et al., 1976; Suresh, 2021). Considering that alveolar type II (AT II) epithelial cells and fibroblasts are often located in close proximity at the periphery of alveolar sacs, the use of AT II-like A549 cells is reasonably justifiable for obtaining *P*_*app*_ values of the epithelial ASO "shielding" (Lieber et al., 1976).

Our biokinetics study through A549 cells revealed that only *ca.* 90% of the apically applied ASO reached the basal medium - *ca.* 10% was retained by the cells. Moreover, the transport kinetics though A549 cells is much faster for nebulized as compared to pipetted ASO delivery (Fig. 5D). This well-known faster drug transport for aerosolized drug delivery in inhalation therapy is often referred to as "burst-like" biokinetics (here to fibroblasts) or "burst-like" pharmacokinetics in the context of inhaled drugs being transported into the blood. This feature is particularly relevant for liquid (not dry powder) drug formulations, since limited powder dissolution in the lung lining fluid may mitigate the pharmacokinetics of dry powders (Meindl et al., 2015). For further investigation of this issue, the widely used concept of apparent permeability *P*_*app*_ was leveraged. According to Eq. (1a), *P*_*app*_ is the Cy5-ASO transport rate into the basal compartment (here: mol/s) normalized to the transwell insert area and the initial molar concentration in the apical compartment (here: 430 μM and 30 μM Cy5-ASO delivered in 7 μL (nebulized) and 100 μL (pipetted), respectively (14- fold different concentration)). The derivation of this equation assumes that the transported dose fraction is relatively small, typically less than *ca.* 25% of the initially applied dose. This requirement is satisfied for the first time point (0.25 h) after pipetted ASO application yielding 23.1 ± 2.1% ASO (mean ± SD) (for *n* = 3: 2 × SEM (95% CL) = 12.9%-rel.; this is also the 95% CL of the measured *P*_*app*_) in the basal medium. Since for nebulized delivery even the dose at the first measured time point gravely exceeded this threshold (75.6% at 0.25 h) the corresponding time point for nebulized application was determined from exponential curve fitting and extrapolation down to the 23.1% basal ASO dose (Fig. 5D). This revealed the 95% confidence band (and mean) of *t*_*23.neb*_ as 54–165 s (121 s) with the mean *t*_*23.neb*_ value being between a factor of 5.5–16.5 (7.4) smaller than that for pipetted delivery (*t*_*23.pip*_ = 0.25 h = 900 s). It is important to note that the relatively large 95% confidence band of *t*_*23.neb*_ is due to the complete lack of dose data between zero and the plateau level of 83.6% (Fig. 5D). With these *t*_*23*_ values, the 95% CL (mean) of *P*_*app*_ can be calculated from Eq. (2) as *P*_*app.neb*_ = 2.1–6.4 (2.9) × 10^−6^ cm/s and *P*_*app.pip*_ = 5.5 (± 0.7) × 10^−6^ cm/s for nebulized and pipetted application, respectively. Thus, both *P*_*app*_ values are identical within experiment uncertainties, *i.e.* the barrier tightness of the A549 cells was independent of the route of application. An overview of these *P*_*app*_ values is given in Table 2. The range of *P*_*app*_ values reported for our ASO is virtually identical to the range of *P*_*app*_ values of 2.9–6.5 × 10^−6^ cm/s reported in the literature for *ca.* 6 kDa fluorescently labelled dextran (FITC dextran), where we averaged the values given for 4 kDa and 10 kDa FITC dextran (Doryab and Schmid, 2022). Thus, ASO permeability through A549 cells is consistent with paracellular transport of dextran molecules of the same molecular mass. This lends credibility to the data presented here and to the important finding that the route of delivery does not affect *P*_*app*_ values. The latter was expected, since the A549 cells tend to respond similarly under submerged and ALI conditions due to lack of strong polarization under ALI conditions (Lenz et al., 2014).

The reasons for the burst-like biokinetics profile for nebulized large are not directly evident. One might have suspected that the tightness of the A549 cell barrier may depend on delivery conditions. Yet this is not the case, since the analysis above has shown that the *P*_*app*_ values and thus the A549 barrier integrity was independent of the route of delivery. One might expect that delivery route dependent difference in ASO concentration or volume of the apically applied liquid could account for this. For constant dose application as in our case, the ratios of both initial apical concentration and of applied dose for the two delivery routes are 14.3 but inverse. According to Fick's law a larger initial apical concentration (*C*_*0*_) will induce a larger mass transport rate. For exploration of this effect of *t*_*23*_ (or any other characteristic time), the 1D diffusion transport equation was solved computationally for the pipetting condition assuming 10- fold lower and 10-fold higher concentrations (*C*_*0*_) in the apical compartment than used here (*C*_*0*_ = 30 μM). The three concentration profiles along the centerline of the transwell insert setup (x-axis in Fig. S6B) show a very similar shape but shifted 10-fold up or down relative to the profile for *C*_*0*_ = 30 μM (Fig. S6B; here: profiles at *t*_*80*_ = 1581 s are depicted). After normalization to the respective *C*_*0*_ values, all three concentration profiles collapsed onto the same profile as depicted in Fig. S6C (black line). However, the shape of the normalized concentration profile depends on time point. For *t* = 0 (*t*_*0*_), the initial apical concentration is uniform (for *C*_*0*_ normalized dose profiles) and zero in the rest of the domain. At the latest timepoint *t*_*infinite*_ the concentration is constant at 0.077 over the entire domain, where the asymptotic concentration for diffusion equilibrium is defined by the ratio of the apical (0.1 mL) and total liquid volume (0.077 = 0.1 mL / (0.1 + 1.2 mL); basal volume: 1.2 mL). In between these two extreme time points, there is a gradual shift of dose from the apical to the basal compartment from *t*_*0*_, *t*_*30*_, *t*_*80*_, to *t*_*infinite*_ as depicted in Fig. S6C. From this it is concluded that albeit the molar (or mass) ASO transport rate (in mol/min) and the invested dose scales linearly with *C*_*0*_ which implies that the dose-normalized transport rate (% of total dose per min) is independent of *C*_*0*_. The same general result is true for the nebulized application as well, but the transport kinetics is faster.

With *P*_*app*_ and *C*_*0*_ having been ruled out, the effect of apical volume on the speed of transport drug transport across a cell barrier can be elucidated with our computational model. For reasons explained below we have to expect considerable uncertainties associated with our model (or any purely diffusional model). Thus, we determined its accuracy by extracting *P*_*app*_ from it for pipetted delivery, for which an accurate empirical *P*_*app*_ values is available, by iteratively changing the ASO diffusion coefficient *Diff*_*cell*_ in the A549 cell layer only (10 μm thick according to (Ren et al., 2016)) until the model-derived basal dose matches the measured basal dose of 23.1% for a transport time *t*_*23*_ = 900 s. The obtained *Diff*_*cell*_ of 1.4 × 10^−8^ cm^2^/s yields a *P*_*app.pip-model*_ = 1.4 × 10^−5^ cm/s, which is 2.5-fold larger than the empirically determined value of *P*_*app.pip*_ = 5.50 × 10^−6^ cm/s. An overview of the measured and computationally modeled *P*_*app*_ values include the key parameters used for calculations is given in Table 2. This 2.5-fold larger *P*_*app.pip-model*_ value can be rationalized by acknowledging the following uncertainties in our model: 1) The estimated ASO diffusion coefficient (in water or media) is likely too large, since biomolecules like ASOs tend to form protein coronas (cell media contains proteins) which enhances their effective molecular weight leading to a smaller diffusion coefficient, which results in a too large *P*_*app.pip-model*_ value, 2) Any pure diffusion model cannot capture biological mechanisms hindering transport kinetics such as P-glycoprotein efflux and the presence of glycocalyx around the cells (Jorgensen et al., 2025; Balbisi et al., 2026), 3) There is always some uncertainty due to MatLab solver of differential equations and due to the projection of the cylindrically 3D symmetric geometry of the insert assay onto its centerline (1D geometry). The combined effect of this is likely negligible compared to the former two aspects, since its magnitude was estimated to be 15% by comparing the ratio of the modeled *t*_*23*_ values for nebulized and pipetted delivery for the membrane without cells yielding a ratio of 12.2, which is 15% lower than the expected ratio of 14.3 (being the ratio of the apical volume for pipetted and nebulized application). Taken together, the observed 2.5- fold deviation between the computationally and experimentally determined *P*_*app.pip*_ value is still within the expected accuracy limit (Table 2, footnote 2).

It is important to note that this 2.5-fold bias in *P*_*app.pip*_ can be corrected for by reducing the ASO diffusion coefficient by a factor of 2.5 (from 1.68 to 0.66 × 10^−6^ cm^2^/s). As the likely reasons for this bias (ASO protein corona, cell biological mechanisms, computational modeling uncertainty) are independent of the apical volume, the computational model can be used to explore the relative effect of apical volume on dose-normalized transport rate through A549 cell. For the following investigation of this issue all modeled parameters are corrected for this 2.5-fold bias. The calculated *t*_*23*_ values for an apical volume of 100 μL (pipetted) and 7 μL (nebulized) yielded 900 s and 63 s. The ratio of these two *t*_*23*_ values is 14.3, which is identical to the ratio of the apical volumes for pipetted and nebulized delivery (Table 2). Taken together, we conclude that the low applied apical volume for nebulized application is the governing factor for the observed burst-like biokinetics profile. As caveat we add that the apical volume-related burst-like transport profile may be altered due to non-linear biological processes such as transporter-molecule dominated transcellular transport which is characterized by reduced transport rates above a certain threshold drug concentration (or dose). It is also important to note that albeit A549 cells are not a suitable model for tight alveolar air-blood barriers, the computational analysis of the underlying reasons for burst-like biokinetics is also valid for drug transport to the blood (pharmacokinetics), since for purely diffusional transport as seen here the tightness of the barrier model does not affect the apical volume-dependent steepness of the temporal transport profile.

**Table 2:** Summary of measured and computationally modeled biokinetics parameters. Where appropriate, the 95% confidence interval and its mean value (in parenthesis) are provided.

Parameters	Pipetted	Nebulized	Ratio (pip/neb)
Measured parameters			
Apical volume (μL)	100	7	14.3
*t*_*23*_ (s)	900	54–165 (121)	5.5–16.5 (7.4)
*t*_*23*_ (s) – apical volume-scaled	900	63^1)^	14.3

Calculated parameters			
*P*_*app*_ (10^−6^ cm/s)	4.8–6.2 (5.5)	2.1–6.4 (2.9)	2.3–1.0 (1.9)
*P*_*app*_ (10^−6^ cm/s) – using apical volume-scaled *t*_*23*_	5.5	5.5	1

Computationally modeled values			
*Corrected P*_*app*_ (10^−6^ cm/s)^2)^	5.5	5.5	1
*t*_*23*_ (s)	900	63	14.3

^1^*t*_*23.neb*_ = 63 s is calculated from *t*_*23.neb*_ = 900 s divided by 14.3, where 14.3 (= 100 μL / 7 μL) is the ratio of the apical volumes for pipetted and nebulized application.

^2^ For reasons explained in the text, the computational model provided 2.5-fold too large *P*_*app*_ values, which was corrected by a 2.5-fold reduction of the effective diffusion coefficient of the ASO.

Considering that *P*_*app*_ is independent of the type of delivery, it is instructive to consider the advantages and disadvantages of performing *P*_*app*_ measurements *via* pipetting or nebulization. Typically, nebulized drug delivery is more time consuming, technically complex and experiences larger data variability mainly due to the Cloud MAX associated CV of 8 to 11%-rel. for repeated ASO (Fig. 4C). The latter is evident from Fig. 5D, especially in the plateau levels above 80% basal dose, where the observed CV is 2.8% (*n* = 2) for pipetting and 9.1 ± 1.9% (*n* = 4) for nebulization (Fig. 5D). The latter is consistent with the expected CV for repeated apical dosing *via* nebulization. These CV values will also propagate into the reported *P*_*app*_ values, *i.e.* the CV of *P*_*app.neb*_ is 9.1 ± 1.9% and that of *P*_*app.pip*_ is 2.8%. On the other hand, for some drugs the nebulization process may affect the molecular structure of the drug and thus its diffusion coefficient, toxicity, and efficacy. Moreover, some *in vitro* cell models may experience loss of polarization and thus barrier tightness when exposed to larger amounts of liquid on the air side. Thus, *in vitro* testing of nebulized drugs may result on clinically more relevant values. On a more general level, we acknowledge that the use of a Cy5-esther fluorescence label (ASO: 5.7 kDa, Cy5-ASO: 6.1 kDa), which increased the molecular weight of the ASO by 7.8% is expected to decreases the effective diffusion coefficient of the ASO and thus the measured *P*_*app*_ by 2.6% (= (7.8%)^1/3^, see Eqs. S2, S3a, S3b) or from the molecular-weight dependent *P*_*app*_ data for A549 cells in (Frost et al., 2019).

Another important aspect of this study is the question if it is recommendable for testing of drugs directed to fibroblasts, which are not directly accessible on the apical side, to use cocultures of these target cells and epithelial cells. In principle, the delayed and reduced ASO delivery to fibroblasts through pipetting administration or shielding of fibroblasts by A549 cells could result in lower ASO efficacy. Since even the delay in LIMCH1-ASO transport for pipetting is relatively short (less than 4 h for reaching asymptotic >80% dose levels (Fig. 5D) compared to the 7-day ASO-cell incubation time, one might not expect a substantial difference in LIMCH1-ASO silencing (efficacy) depending on the application route for A549-cocultured fibroblasts as compared to monocultured fibroblasts. In fact, a significant effect of delivery route could only be observed for the lower dose of 3 nmol in the coculture model (Fig. 6C). However, for both doses *LIMCH1* silencing was more effective in monocultured (non-A549 shielded) fibroblasts, suggesting some kind of dose “shielding” by A549 cells independent of the ASO delivery route (Figs. 6B, and C). It is not likely that the observed *ca.* 10% dose shielding by A549 cells can account for a rather substantial (2- to 4- fold reduction LIMCH1 silencing). Considering that the 8–11% variability in repeated dosing by the Cloud MAX does not induce a significantly larger variability in LIMCH1 silencing as compared to pipetted application, it is more likely that the presence of A549 epithelial cells enhances the resilience of the primary fibroblasts of IPF patients against LIMCH1 silencing by the ASO. An effect of the burst-like ASO biokinetics profile on ASO efficacy was only observed for low ASO dose (3 nmol), which is intuitively expected to be more sensitive to biokinetics than the larger dose. On the other hand, under clinical conditions the alveolar epithelial barrier is expected to be much tighter than our A549 cell model. Thus, it is conceivable that under clinical conditions epithelial dose shielding may have an effect on ASO efficacy in fibroblasts.

The translational value of the ASO efficacy study can be explored by comparing our *in vitro* data with *in vivo* ASO dose-response relationships in animal models. A recent study showed that intratracheal instillation of ASO into the lungs of mice resulted in approximately 2- fold downregulation of Malat1 IncRNA in lung tissue between 6 h and 28 d after the application of 7.9 pmol/cm^2^ Malat1 LNA ASO, a gapmer ASO with locked nucleic acid ribose chemistry (Bäckström et al., 2024). The *in vivo* dose was calculated based on the provided dosing information (0.19 nmol/g) and body weight (25 g), as well as the typical epithelial surface area of the murine lung (600 cm^2^) (Schmid and Stoeger, 2016). The *in vitro* experiments reported here were performed with a low dose of 640 pmol/cm^2^ (3 nmol per insert), resulting in 5- to 12-fold RNA downregulation for the co- and monoculture model, respectively, at seven days after ASO treatment. In *in vivo* settings the LNA ASO downregulation is 2.5- to 6.5-fold less than for *in vitro* conditions at seven days after ASO delivery, but at a *ca.* 100-fold lower tissue dose. However, ASO efficacy strongly depends on several parameters including the chemistry of the ASO, the degree to which the respective target gene is upregulated, the number of target cells that express this gene, and the location of these target cells relative to the drug distribution profile in lung tissue. Despite these uncertainties between the Malat1 LNA ASO and our LIMCH1-ASO, there is a general consistency between the *in vitro* data presented here and the *in vivo* mouse data reported by Bäckström et al.*,* study (Bäckström et al., 2024). To the best of our knowledge, there is no *in vivo* animal or patient data on ASO biokinetics through the epithelium into the lung tissue. Yet, our *in vitro* efficacy and dose shielding study suggests that epithelial shielding may have the potential to reduce ASO efficacy in subepithelial target cells - at least for acute responses. Moreover, due to the to the limited lifetime of A549 cell model (up to *ca.* 10 days) (Barosova et al., 2021), our data are likely not predictive for long-term repeated ASO dosing as required for the treatment for chronic diseases.

### 4.3 Clinical relevance and regulatory approval

One of the main criteria for regulatory approval of *in vitro* assays as presented here is their clinical relevance and validation. This section addresses the clinical relevance of the Cloud MAX technology, with particular focus on aerosol delivery characteristics, ASO efficacy, and bio−/pharmacokinetic considerations. A few more aspects on regulatory approval are addressed at the very end of this section.

The Cloud MAX delivers drug doses comparable to those obtained in clinical settings. To assess the clinical relevance of the Cloud MAX technology, it is instructive to consider that inhalation therapy typically yields drug deposition efficiencies of 10–40%. In other words, up to 40% of the inhaled drug/aerosol dose is deposited in the lungs (Lavorini, 2013). Consequently, high-performance nebulizers with an output rate of up to 1 mL/min and breath-activated aerosol generation ensures that as little as possible of the aerosol is “wasted” during the exhalation phase which is typically *ca.* twice as long as the inhalation phase (for spontaneous breathing). Even with breath activation only about 50% of the invested drug reaches the mouthpiece of the inhaler. Thus, a maximum aerosol volume of 4 mL (equalling 1 mL/min × 20 min × 40% × 50%) is deposited in the lungs during 20 min of inhalation therapy (the maximum tolerable time for patients). Considering the approximate 100 m^2^ of lung epithelium, this yields a surface-specific aerosol dose of 4 nL/cm^2^ corresponding to a 40 nm thick liquid layer on the epithelium and an average dose rate of 0.2 nL cm^−2^ min^−1^. The performance analysis of the Cloud MAX showed that the least amount of liquid deposited on the cells is 1.1 μL after nebulization of 3 μL, with a fractional deposition efficiency of 0.36 (Fig. 3A, 40 mm exposure tube). For 6-well transwell inserts with an area of 4.7 cm^2^, this corresponds to a surface-area normalized volume dose of 240 nL/cm^2^ (*i.e.* a 2.4 μm thick liquid layer is covering the epithelium). Thus, the Cloud MAX delivers surface-specific doses that are at least 60 times larger than the spatially averaged dose in patients. However, aerosol deposition in the lungs is not uniform, and hot spot regions are known to occur in bifurcations of the bronchial tree and the central alveolar duct in the proximal acinar region during inhalation therapy with dose enhancement factors ranging from 40 to 350, depending on the region, airflow rate, and aerosol size (Yang et al., 2024; Schmid and Stoeger, 2016). Thus, the Cloud MAX tissue-specific dose represents the conditions in the hot spot dose regions of the lung, which have been speculated to be the drives of biological effects such as particle toxicity (Shankar et al., 2025; Tran et al., 2000). Moreover, from these considerations it is evident that the *in vitro* dose rate is even at least 600- fold larger than in clinical settings (Cloud MAX: 120 nL cm^−2^ min^−1^; clinical: 0.2 nL cm^−2^ min^−1^), since the time for delivery of the *in vitro* dose is with 2 min (in exposure tube) 10-fold lower than in our clinical exposure scenario (20 min). The effect of dose rate on drug efficacy has not been investigated very much, but slow dose rate leads to longer exposure time and thus more time for mucociliary or macrophage clearance of the drug potentially resulting in reduced efficacy. As there is typically no clearance for *in vitro* conditions (at least in the absence of macrophages), it can be expected that the effective dose under clinical conditions is lower than the dose calculated above, *i.e.* the effective *in vitro* to pulmonary retained clinical dose ratio is larger than 60. The entire discussion above was conducted with respect to tissue deposited drug volume, *i.e.* we assumed that the drug mass concentration in the nebulized drugs was identical. Moreover, it is important to recognize that by selecting the lowest possible *in vitro* dose and the highest achievable clinical dose, we have calculated the lowest *in vitro* to clinical dose ratio. Considering that one can easily increase the *in vitro* nebulized volume by a factor of 10 and reduce the clinically nebulized volume by a factor of 100 (*e.g.* by using pressurized metered dose inhalers rather than nebulizers), the effective *in vitro* dose can exceed 10^5^- fold the clinically relevant dose. Depending on the type of drug, its mode of action and the choice of *in vitro* model, the clinical dose-specific drug efficacy may be larger or smaller than for *in vitro* conditions. Although advanced *in vitro* lung models (*e.g.*, ALI cell cultures) increasingly reproduce key aspects of human pulmonary physiology, their clinical validation for predicting efficacy of inhaled drugs remains limited. While certain *in vitro* systems can achieve quantitative *in vitro*-*in vivo* correlations for pharmacokinetic endpoints, the relationship between *in vitro* exposure and clinical therapeutic response is still poorly defined due to variability in lung deposition, patient-related factors, and challenges in measuring local drug concentrations *in vivo (*Fröhlich*, 2019**;* Metz et al., *2022**)*. Thus, it is useful to have both *in vitro* aerosol-cell delivery systems which exceed the clinically relevant dose and *vice versa*. The former is accomplished with the Cloud MAX and the latter can be accomplished with the Cloud MAX by using lower drug concentrations than in clinical settings and / or by further reducing the amount of nebulized drug.

This *in vitro* study has clearly demonstrated that the burst-like bio−/pharmacokinetics profile, which has often been reported for nebulized *in vitro* cell models, can be explained by the small apical drug dose delivered during inhalation therapy (of cells). Albeit this is also expected to be correct for clinical conditions due to the underlying physical concepts being independent of the specific conditions. To the best of our knowledge no clinical studies have been performed yet to confirm this assumption. This notion has been indirectly corroborated by the fact that clinically observed pharmacokinetics profiles can be predicted reasonably well based on *in vitro P*_*app*_ values (Sakagami, 2020). However, in most cases the actual transport rates from the apical into the blood compartment cannot be predicted directly from *in vitro P*_*app*_. Often *in vitro* / *ex vivo* (perfused lung) or *in vitro* / *in vivo* (animal) correlations of *P*_*app*_ with observed transport rates are required for robust quantitative predictions of clinically observed pharmacokinetics profiles (Sakagami, 2020). Importantly, this study has demonstrated that the observed burst-like transport rates observed for nebulization are not affecting the resulting *P*_*app*_ value. As caveat we add, that unlike A549 cells, which have been shown to behave very similar under submerged and at ALI conditions (see present study and (Lenz et al., 2014)), most epithelial cell cultures express a more tight and functional barrier function under ALI conditions due to cell polarization and/or secretion of protective mucus and lung lining fluids (Fröhlich, 2019). Therefore, it is conceivable that ALI-derived *P*_*app*_ values are more representative of *in vivo* bio−/pharmacokinetics than those derived from submerged cell culture models.

Considering the specific case of ASO nebulization of 20 μL with the Cloud MAX, the thickness of the liquid layer was 15 μm (1.5 μL/cm^2^ = 7 μL per 4.7 cm^2^ of cells). This layer is approximately 200 times (=15 μm / 80 nm) thicker than the layer formed during inhalation therapy with 20 mL of drug as described above, where we considered that in addition to the aerosol deposited liquid layer (40 nm thickness) there is also a *ca.* 40 nm thick layer of alveolar lining fluid present during inhalation therapy. It is conceivable that this excessive amount of liquid (ASO formulation) may exert a certain level of strain to the *in vitro* cultured cells, yet none of our toxicity assays detected any signs of cellular stress. From our analysis of the reasons of the burst-like bio−/pharmacokinetics profile, we concluded that the burst-like transport rate (%-drug transport per time and per tissue area) scales linearly with the apical volume per area. Taking into account the effect of apical volume on transport kinetics demonstrated in the present study, the clinically expected time for transport of 23% of the lung deposited ASO dose to reach the fibroblasts in the interstitial alveolar tissue within less than 0.3 s (= *ca.* 63 s/200; with *t*_*23*_ (*in vitro*, nebulized - computationally modeled value, Table 2) = 63 s). On the other hand, it is likely that the alveolar epithelial barrier tightness in patients is larger than that of the A549 cells (alveolar type II-like epithelial cell). As an upper limit of this tightness, we can assume *P*_*app*_ = 0.02 × 10^−6^ cm^2^/s as one of the highest values reported in the literature for primary alveolar type I epithelial (ATI) cells (for 4–10 kDa FITC-dextran) (Doryab and Schmid, 2022). Considering that *P*_*app*_ (A549) = 5.5 × 10^−6^ cm^2^/s for Cy5-ASO (6.1 kDa) (Table 2), this yields a 275-fold retardation of ASO transport resulting in *t*_*23*_ = 82 s (0.3 s × 275). Thus, due to the compensating effects of even smaller apical volume and enhanced barrier tightness, the *in vitro t*_*23*_ (A549) value of 63 s is very close to the expected clinical *t*_*23*_ of 82 s. As caveat we add that clinical value this is only a rough estimate, since permeability may not only dependent on the molecular weight but also on the molecular structure of the drug. For assessment of the actual dose shielding effect in clinical settings, one would not only need to assess permeability of the epithelial barrier, but also ASO uptake and bioactivity in fibroblasts. The latter information is neither available for *in vitro* nor for clinical conditions. Interestingly, since ATI cells are representing the air-blood barrier of this lung one can use the ratio of *P*_*app*_ for A549 and ATI to infer the ATI equivalent *in vitro t*_*23*_ value ibeing 4.8 h (= 63 s × 275) and 69 h (= 900 s × 275) for nebulized and pipetted application, respectively. Especaially, the latter is exteremly slow and sometimes even exceeds the "lifetime" of *in vitro* cell culture models during which tight barrier function is maintained.

Translation of the efficacy (therapeutic) window of *in vitro* assays to clinical settings is currently not well established. It depends on numerous parameters – many of them not known such as *in vitro* model sensitivity relative to clinical conditions, metabolic degradation, and cell-specific drug dose and bioactivity. Thus, here we only state that *in vivo* mouse studies have reported efficacy of a Malat1 LNA ASO at 7.9 pmol/cm^2^ in a mouse model (Bäckström et al., 2024), which is *ca.* 100-fold lower than our lowest effective dose level of 640 pmol/cm^2^, but we have not explored the *in vitro* sensitivity towards lower doses. It is not unusual to observe two to three orders of magnitude difference between *in vitro* model sensitivity and sensitivity under *in vivo* (animal) or clinical conditions, and this deviation has been observed in both directions depending on the type of drugs and types of cell model (Hickey et al., 2024).

We acknowledge that aerosol-based drug testing may not be feasible in early preclinical *in vitro* studies, especially when formulation development is still substantial, the inhaler is not yet defined, or suitable aerosol-cell exposure systems like the one presented in this study are unavailable. Nevertheless, the incorporation of bioactivity (toxicity, efficacy) and absorption (here: biokinetics, as part of ADME testing) into early preclinical testing may enhance the accuracy and clinical relevance of preclinical drug testing. Importantly, with the availability of medium throughput technologies for expediting the design of inhalable drug formulations without requiring nebulization (Röhm et al., 2017) and technologies for dose-controlled aerosol-cell exposure with clinically relevant dose rates such as the ALICE Cloud MAX, *in vitro* cell-based drug testing with nebulization has come within reach even for early *in vitro* drug testing (Rittersberger et al., 2025).

Finally, the Cloud MAX-based *in vitro* assay presented here for an ASO targeted to alveolar fibroblasts can support the clinical translation of preclinically tested drugs by contributing to the establishment of a well-defined Target Product Profile (TPP). The TPP is a strategic planning document that defines the intended characteristics of a future drug product and serves as a roadmap to guide its development from the preclinical stage through the conduction of clinical trials and subsequent regulatory approval for a specific medical need (WHO.int). The assay description presented here can provide guidance on 1) experimental design of *in vitro* efficacy and safety assays for inhaled drugs, 2) dosing and administration (nebulization) of drugs, 3) dose-dependent efficacy with respect to target engagement (here: downregulation of LIMCH1 gene expression in lung fibroblasts), 4) dose-dependent cytotoxicity, and 5) drug biokinetics through an epithelial lung barrier to fibroblasts and pharmacodynamics in fibroblasts.

## 5 Conclusions

This study has demonstrated that the Cloud MAX is an efficient (up to 50%), dosimetrically accurate aerosol-cell delivery system for microliter-scale *in vitro* testing of inhaled medicine with medium throughput (six inserts within 7.5 min). Its efficiency of substance use is with 52% and 37% for nebulization of 3 to 5 μL and 7.5 to 20 μL, respectively, almost as efficient as pipetting (100%).

The Cloud MAX was designed to mimic the conditions during inhalation therapy including the duration of drug delivery (a few minutes at the most), the amount of inhaled liquid volume which is deposited per area of lung (still 200-fold too large, but 300-fold better than submerged culture conditions) and induced mechanical and thermal strain on the drug during the nebulization process. Albeit aerosol-based drug testing may not always be feasible in early preclinical *in vitro* studies, the incorporation of nebulization and low-volume aerosol deposition may enhance the clinical accuracy of *in vitro* drug testing with respect to bioactivity (toxicity, efficacy) and absorption (here: biokinetics, as part of ADME testing). For guidance on inclusion of nebulization during preclinical *in vitro* drug testing, a clear overview of best-handling practice, advantages and disadvantages of the Cloud MAX technology has been provided.

Collectively, the results presented in this study demonstrate the utility of the Cloud MAX platform for preclinical *in vitro* evaluation of inhaled therapeutics, particularly for - but not limited to - compounds with limited availability or high cost. Moreover, this study demonstrated that even relatively simple computational models can enhance our mechanistic understanding of specific features of *in vitro* pharmacokinetics such as burst-like drug transport profiles.

## CRediT authorship contribution statement

**Pramod Kumar:** Writing – review & editing, Writing – original draft, Visualization, Validation, Project administration, Methodology, Investigation, Formal analysis, Data curation. **Ashesh Chakraborty:** Writing – review & editing, Writing – original draft, Visualization, Formal analysis, Data curation. **Elias Richter:** Writing – review & editing, Visualization, Validation, Methodology, Investigation, Formal analysis, Data curation. **Lin Yang:** Writing – review & editing, Validation, Formal analysis. **Andreas Schröppel:** Writing – review & editing, Validation, Resources. **Coralie Mergault:** Writing – review & editing, Validation, Methodology, Formal analysis, Data curation. **Larissa Rothkirch Née Knüppel**: Investigation, Methodology. **Frank Jaschinski**: Funding acquisition, Resources, Writing – original draft, Supervision. **Marion Blayac:** Writing – review & editing, Formal analysis. **Rita Vanbever:** Writing – review & editing, Supervision, Resources, Funding acquisition, Conceptualization. **Laurent Plantier:** Writing – review & editing, Supervision, Funding acquisition, Conceptualization. **Nathalie Heuzé-Vourch:** Writing – review & editing, Supervision, Funding acquisition, Conceptualization. **Otmar Schmid:** Writing – review & editing, Writing – original draft, Supervision, Resources, Funding acquisition, Formal analysis, Conceptualization.

## Declaration of competing interest

The authors declare the following financial interests/personal relationships which may be considered as potential competing interests:

Dr. Otmar Schmid reports equipment, drugs, or supplies provided by VITROCELL systems (Waldkirch, Germany). The Helmholtz Center Munich (Dr. Otmar Schmid) has licensed the patent for the ALICE Cloud MAX technology (no. WO 2014/147229 A1) to Vitrocell Systems (Waldkirch, Germany). If there are other authors, they declare that they have no known competing financial interests or personal relationships that could have appeared to influence the work reported in this paper.

## Data Availability

Data will be made available on request.
