# Supplementary material for: Microliter-scale in vitro aerosol-cell delivery of inhalable antisense oligonucleotide (ASO) induces “burst-like” pharmacokinetics
Source: Int J Pharm X. 2026 Jul 1;12:100588. doi: 10.1016/j.ijpx.2026.100588 (PMC13743522; doi:10.1016/j.ijpx.2026.100588)
Supplement: Supplementary file 1 [file mmc1.pdf]

Supplementary material

Microliter-scale *in vitro* aerosol-cell delivery of inhalable antisense oligonucleotide (ASO) induces "burst-like" pharmacokinetics

Pramod Kumar [a,1], Ashesh Chakraborty [a,1], Elias Richter [a], Lin Yang [a], Andreas Schröppel [a], Coralie Mergault [b,c], Larissa Rothkirch Née Knüppel [d], Frank Jaschinski [d], Marion Blayac [a], Rita Vanbever [e], Laurent Plantier [b,c,f], Nathalie Heuzé-Vourch [b,c], Otmar Schmid [a,*]

[1]Shared first author

*Correspondence author

Dr. Otmar Schmid

E-mail: otmar.schmid@helmholtz-munich.de

Tel: +49 89 3187-2557

Fax: +49-89-3187-2400

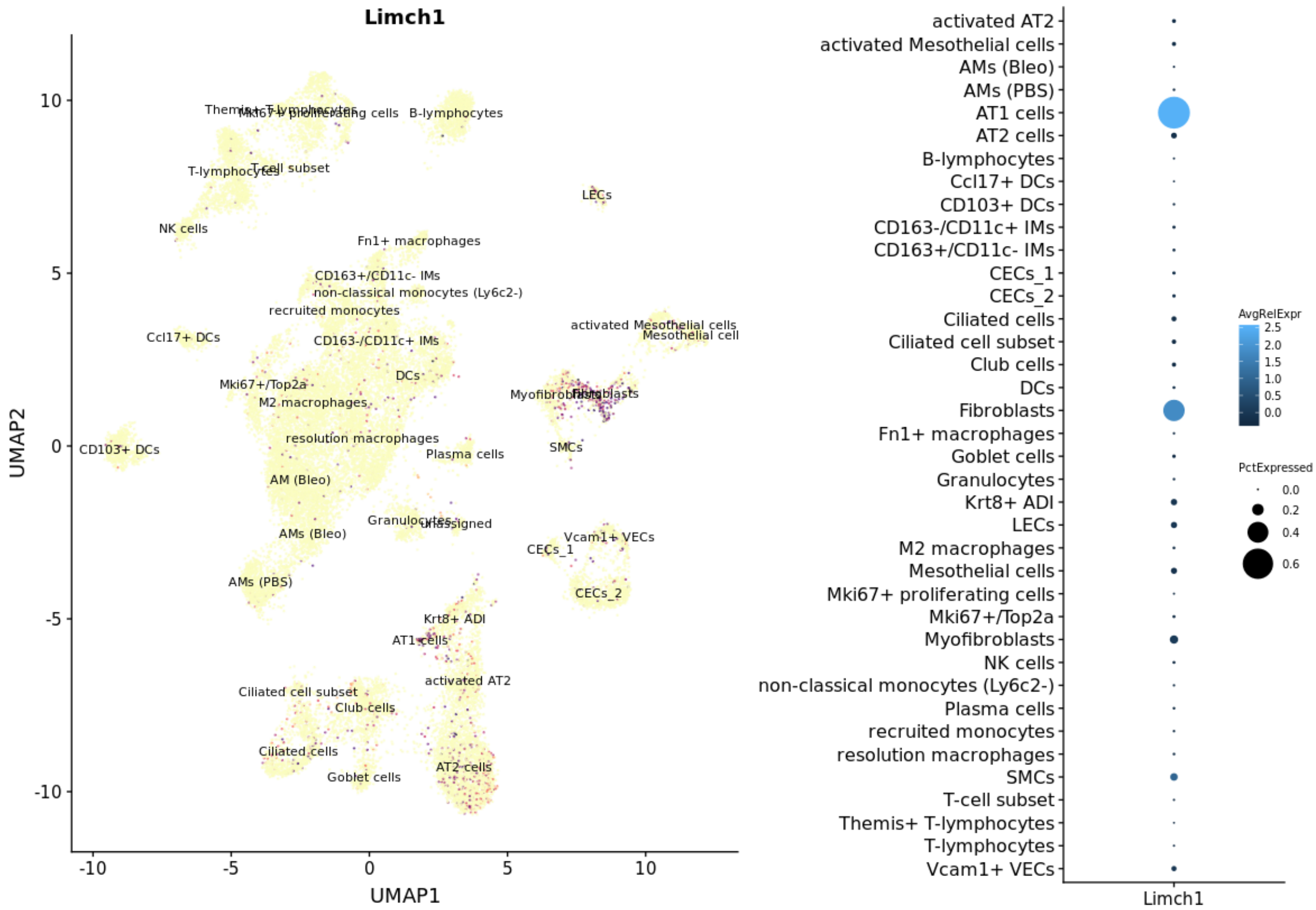

Supplementary figure S1: *LIMCH1* mRNA levels are upregulated in myofibroblasts, fibroblasts, and alveolar type 1 (AT1) cells re-analyzed from single cell RNA sequencing data of bleomycin induced pulmonary fibrosis in mouse lung [1].

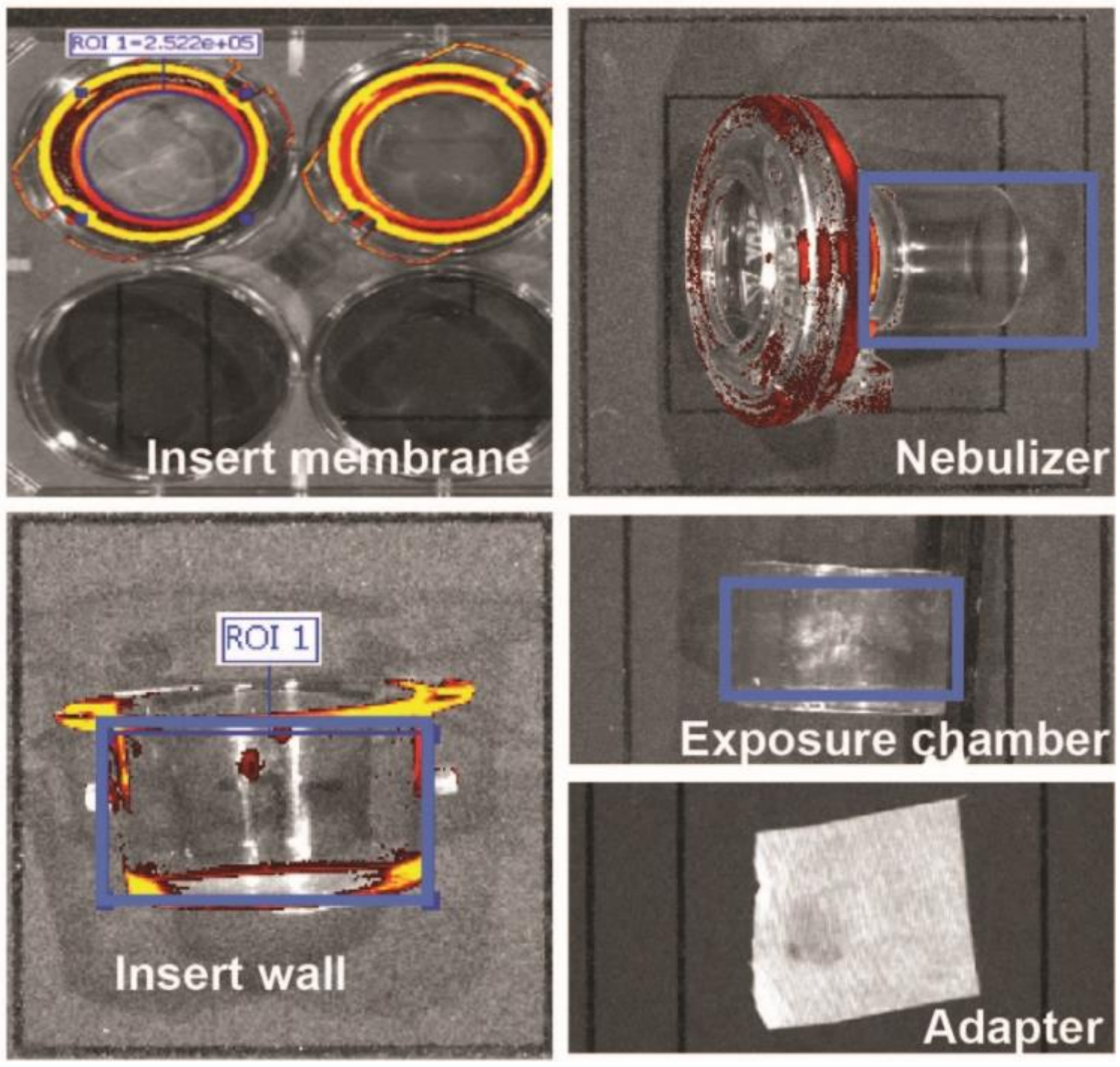

Supplementary figure S2: Qualitative assessment of the blank insert membrane, insert wall, and Cloud MAX device components such as nebulizer, exposure chamber and adapter determined using IVIS system prior to nebulization. The blue rectangles indicate the regions of interest for consideration of the results in the main body of the text.

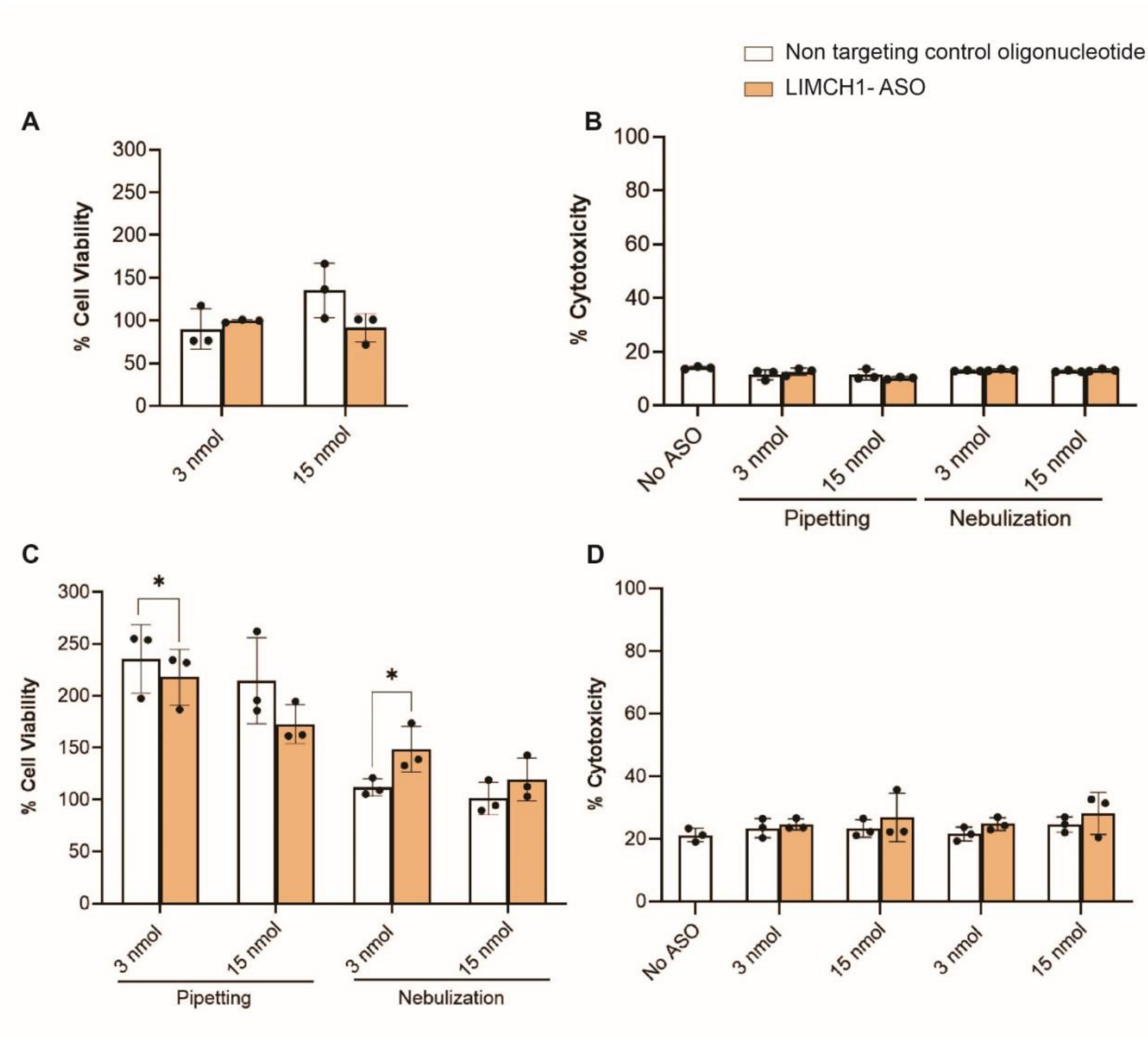

Supplementary figure S3: **Exposure of both monoculture (only IPF-HLFs) and co-culture (A549-IPF-HLFs)** ***in vitro*** **models to ASO is non-toxic. (A)** The percentage cell viability and **(B)** the percentage cell cytotoxicity of IPF-HLFs treated with ASO via pipetting at two different concentrations 3 nmol (i.e., 0.67 nmol/cm^2) or 15 nmol (i.e., 3.21 nmol/cm^2) was determined using WST1 assay and LDH assay, respectively. **(C)** The percentage cell viability and **(D)** the percentage cell cytotoxicity of A549-IPF-HLFs co-cultures treated with ASO either via pipetting or nebulization at two different doses was determined using WST1 and LDH assay, respectively. For the percentage cell viability in **(A)** and **(C)**, the normalization was carried by measuring WST1 activity of IPF-HLFs or A549-IPF-HLFs without any ASO treatment. For the percentage cell cytotoxicity in **(B)** and **(D)**, the normalization was carried out with respect to total LDH obtained by lysing the cells with 1% Triton-X. For figure panel **(A-D),** the statistical analysis was performed using paired student *t*- test. Significance shown when *p*- value < 0.05 (*).

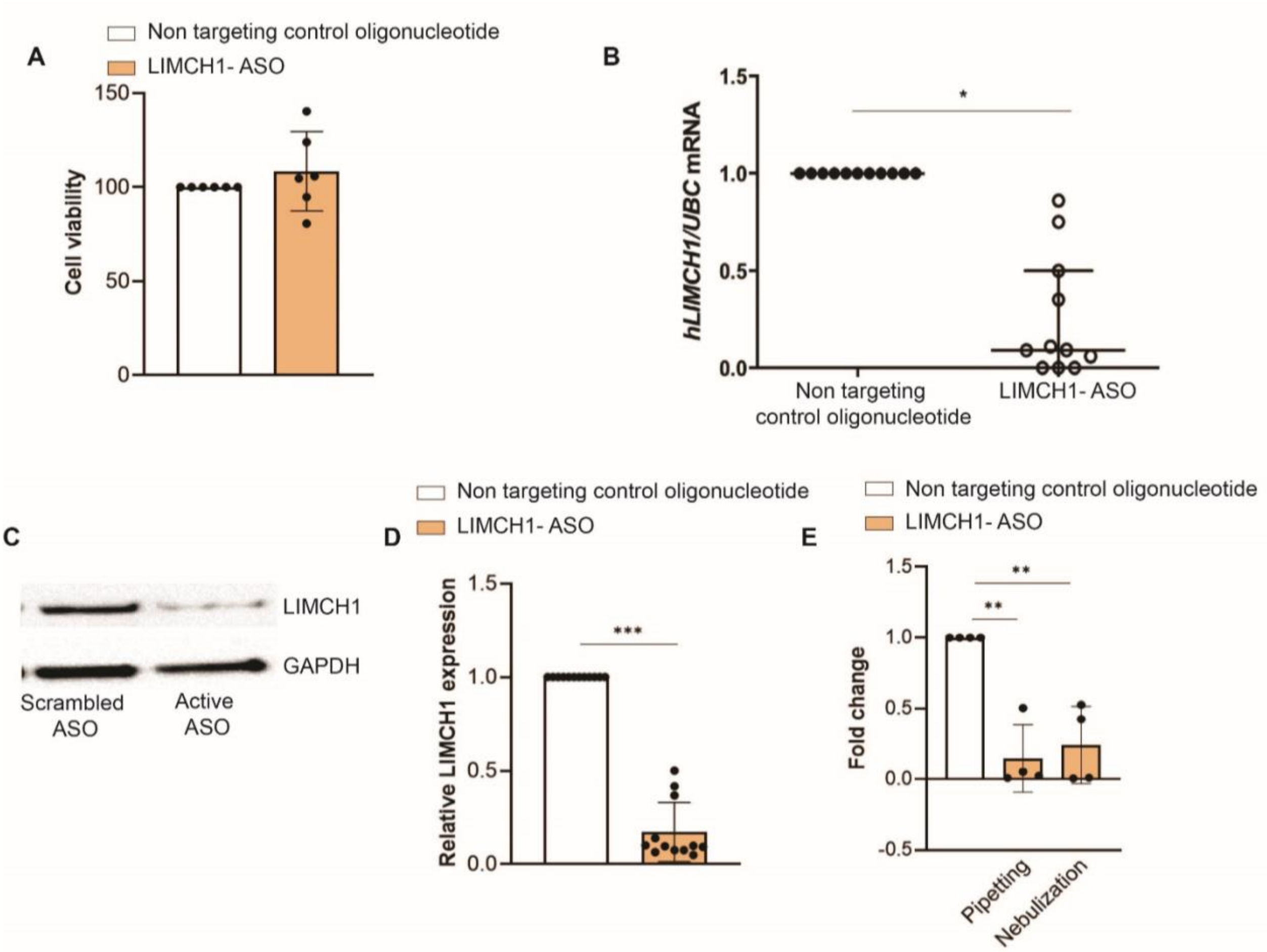

Supplementary Figure S4: LIMCH1 ASO treatment does not affect cell viability and effectively downregulates human *LIMCH1* in CCD-16Lu human lung fibroblasts. (A) Cell metabolic activity in CCD-16Lu cells was assessed with the MTS reagent as a marker for cell viability after 3-4 days of 1 μM (i.e., dose of 0.29 nmol/cm^2) non-targeting control oligonucleotide or LIMCH1-ASO treatment. Results were expressed relative to non-targeting control oligonucleotide condition. Data presented as mean ± SD (n=6). **(B)** qRT-PCR analysis of human *LIMCH1* transcript in CCD-16Lu cells treated with 1 μM ASO (i.e., dose of 0.29 nmol /cm^2) for 3 days. Data presented as mean ± SD (n=3). Transcript analysis of human Ubiquitin C (*UBC*) was used as an internal reference. **(C)** Western blot analysis and **(D)** related quantification of the human LIMCH1 protein in CCD-16Lu cells treated with 1 μM ASO (i.e., 0.26 nmol/cm^2) for 3 days. Data presented as mean ± SD (n=12). **(E)** Efficacy of LIMCH1-ASO is preserved after nebulization. qRT-PCR analysis of human *LIMCH1* transcript in CCD-16Lu cells treated under submerged conditions with ASO either after nebulization or prior to nebulization (pipetting) at a concentration of 1 μM (i.e., 0.29 nmol/cm^2). Data presented as mean ± SD (n=4). Transcript analysis of human Ubiquitin C (*UBC*) was used as an internal reference. The statistical analysis for figure **(A, B, and D)** was performed using nonparametric Wilcoxon paired *t*-test. For figure **(E)**, the statistical analysis was performed using one way ANOVA with Holm-Sidak's multiple comparisons test. Significance shown when *p*- value < 0.05 (*), *p*- value < 0.01 (**), and *p*- value < 0.001 (***)**.**

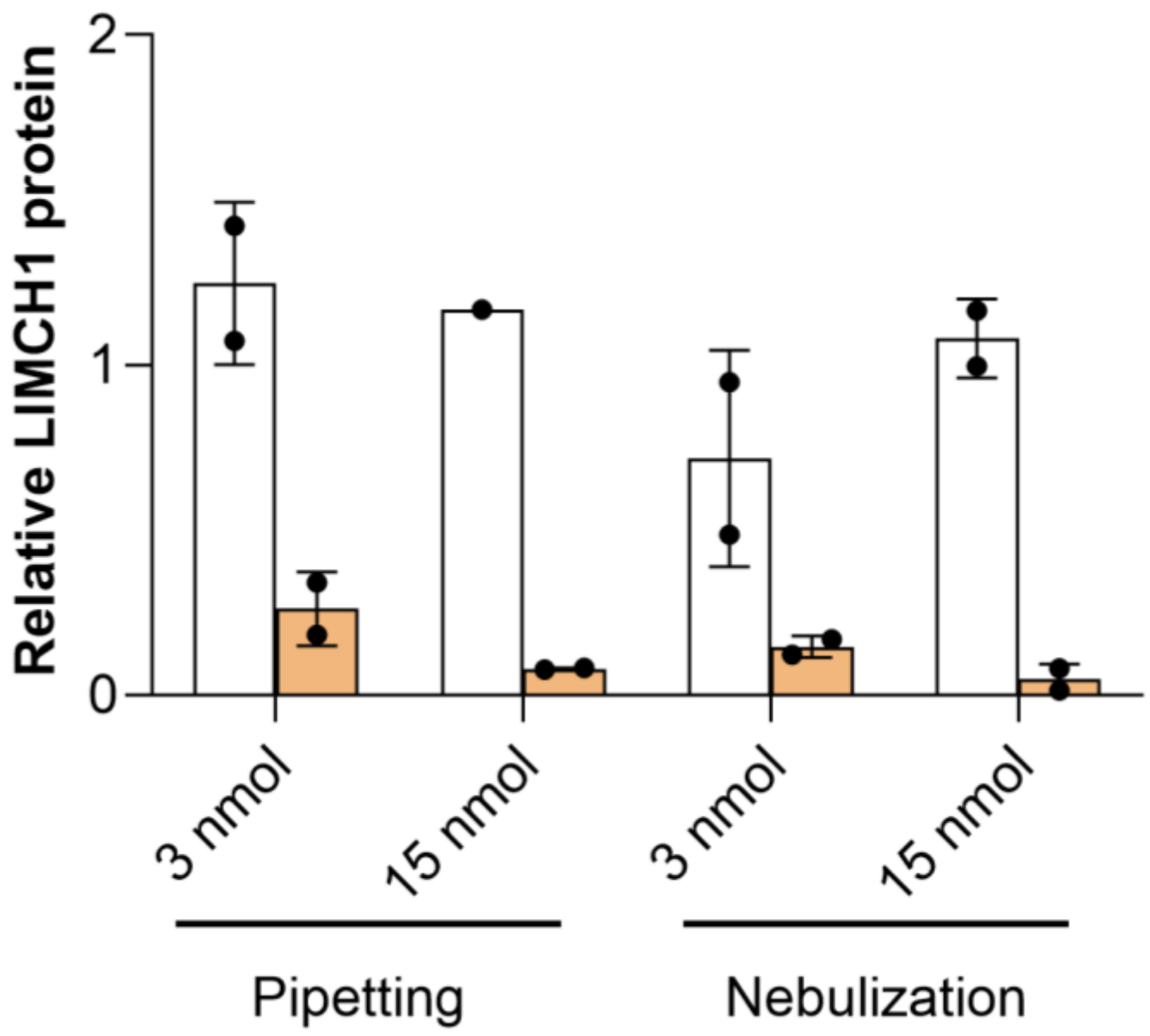

Supplementary figure S5: LIMCH1-ASO treatment inhibits LIMCH1 protein level in *in vitro* co-culture model comprising of A549 cells and IPF-HLFs. Western blot quantification of the human LIMCH1 protein level in the co-culture *in vitro* model observed in primary IPF-HLFs, following LIMCH1-ASO application at two different doses of 3 nmol (i.e., 0.64 nmol/cm^2) or 15 nmol (i.e., 3.21 nmol/cm^2) via either nebulization (7 µL) or pipetting (100 µL). Data are presented as mean$\pm$ SD, n=2.

Detailed description of computational ASO transport model across an *in vitro* cell barrier

Typically, apparent permeability P_{app} of a given drug is measured *in vitro* using a transwell insert setup with relatively similar volume of liquid in the apical (donor) and basal (recipient) compartment. In this case one can assume a time-independent, constant concentration in the donor compartment (C_0), if the concentration in the basal compartment is no more than ca. 25% of C_0. For the present study, the volume in the apical compartment is significantly smaller than that in the basal compartment, i.e. the assumption that C_0 is constant for basal concentrations smaller than 25% of C_0 is not valid anymore. Thus, the standard equation for obtaining P_{app} using a transwell insert setup (see Equation 1a and 1b, Sect. 2.9) is not valid anymore. This limitation can be overcome by computational approaches, which take into account the correct concentration gradient across the membrane at any time point.

In this study, we converted the radially symmetric 3D geometry of the transwell insert setup for P_{app} measurements into a one-dimensional (1D) analogous geometry and computationally solved the 1D time-dependent diffusional transport equation

$$\frac{\partial C(x,t)}{\partial t} = Diff\,\frac{\partial^2 C(x,t)}{\partial x^2}, \qquad \text{(S1)}$$

where *C(x,t)* is the ASO concentration at position *x* and time *t* and *Diff* is the ASO diffusion coefficient (cm²/s).

Conversion of 3D into 1D geometry: The 1D analogous geometry can be visualized as the radially symmetric 3D geometry of the transwell insert setup projected onto its centerline (figure S6A). Only one geometric adaption was necessary due to the apical compartment (insert and membrane) having a smaller radius than the basal compartment (1.22 cm radius versus 1.75 cm radius respectively). Thus, along the 1D centerline each pixel in the apical compartment represents a 2.06-fold smaller volume (2.06 = $(1.75\ \text{cm})^2$/ $(1.12\ \text{cm})^2$) than in the basal compartment. To account for this, the 1.1 mL basal volume with an original cell culture medium height of 1.14 mm was redistributed over 2.4 mm (2.4 mm = 1.14 mm x 2.06) (figure S6A). The 1D transport equation was implemented in MATLAB (version 2025a) and numerically solved using the built-in ode45 solver.

All parameters necessary to solve the 1D diffusion equation are given below:
1) Dimensions of apical, barrier and basal compartments defining the x positions

The zero point of the x-axis was positioned at the top of the apical compartment. In the pipetting condition, applying 100 µL of liquid, the height (length) of the apical donor compartment was 0.21 mm. In the case of the nebulization (7 µL of liquid), the apical length was 0.015 mm. The thickness (length) of the cell-membrane barrier was considered as the sum of the thicknesses from the membrane (10 µm) and that of the confluent A549 cell layer (4 µm) resulting in a total length of 0.014 mm. According to our previous geometric assumption, the volume-adjusted height of the basal recipient compartment was 2.4 mm.

2) Diffusion coefficients

- The 3D diffusion coefficient *Diff* of the ASO molecule (*Diff* = 2.91×10^{-6} cm²/s) in water was calculated from its known molecular weight and a combination of the van-der-Waals (Equation S2) and the Einstein-Stokes equation:

$$Diff = \frac{kT}{6\pi\eta r} \quad \text{(S2)}$$

with k the Boltzmann constant (1.38×10^{-16} cm² g s^{-2} K^{-1}), T the temperature (310.15 K for 37°C), η the dynamic viscosity (0.006913 g $cm^{-1}s^{-1}$), and r the radius of the diffusing molecule (cm).
For 1D models Diff has to be divided by $3^{0.5}$, to account for the fact that motion is occurring only in one direction. Thus, $Diff_{1D} = Diff / 3^{0.5} = 1.68 \times 10^{-6}$ cm²/s.

- The molecular radius r of 2.6 nm was obtained from the molecular mass M of 6151 Da (g/mol) of the Cy5-ASO molecule using the van-der-Waals volume equations:

$$r = \left(\frac{3M}{4\pi\rho NA} \right)^{\frac{1}{3}} \quad \text{(S3a)}$$

$$M = \frac{4}{3}\pi r^3 \rho NA \quad \text{(S3b)}$$

with ρ the density of ASO (1.7 g/cm^3, density of nucleic acids) and *NA* is the Avogadro constant (6.022x10^{-23} mol^{-1}).

- The corresponding ASO diffusion coefficient in the membrane ($Diff_{membrane}$ = 0.238x10^{-6} cm²/s) was estimated from the manufacturer-provided thickness (10 µm) and porosity of the membrane (14.1% for a 4.67 cm² membrane with 3 µm diameter pores and 2x10^6 pores/cm²) as $Diff_{membrane}$ = 0.141 *Diff*.
- The diffusion coefficient of the cell-covered membrane ($Diff_{cell}$) having also a thickness of 10 µm [2] was determined by iteratively adjusting $Diff_{cell}$ to match the measured time t_{23} and t_{80} for transporting 23% (or 80%) of the total dose to the basal compartment. This was done for both pipetted and nebulized application. Averaging over both conditions, we found $Diff_{cell}$ = 1.4 x10^{-8} cm²/s.
- The relationship between P_{app} and $Diff_{cell}$ is given by $P_{app} = Diff_{cell}/h$, where here *h* represents the thickness of the cell layer (or empty membrane) both being 10 µm. Considering the above average $Diff_{cells}$ value, we obtained an average P_{app} = 1.4 x10^{-5} cm/s.

3) Initial and boundary conditions

At time point zero (t = 0 s): The ASO concentration in the apical compartment was C_0; the concentration in the cell-membrane and basal compartment is zero. At the boundaries of the domain: No flux out of the domain is allowed, i.e. $dC/dx = 0$ at x= 0 and x = $h_{Apical} + h_{cell} + h_{Basal}$).

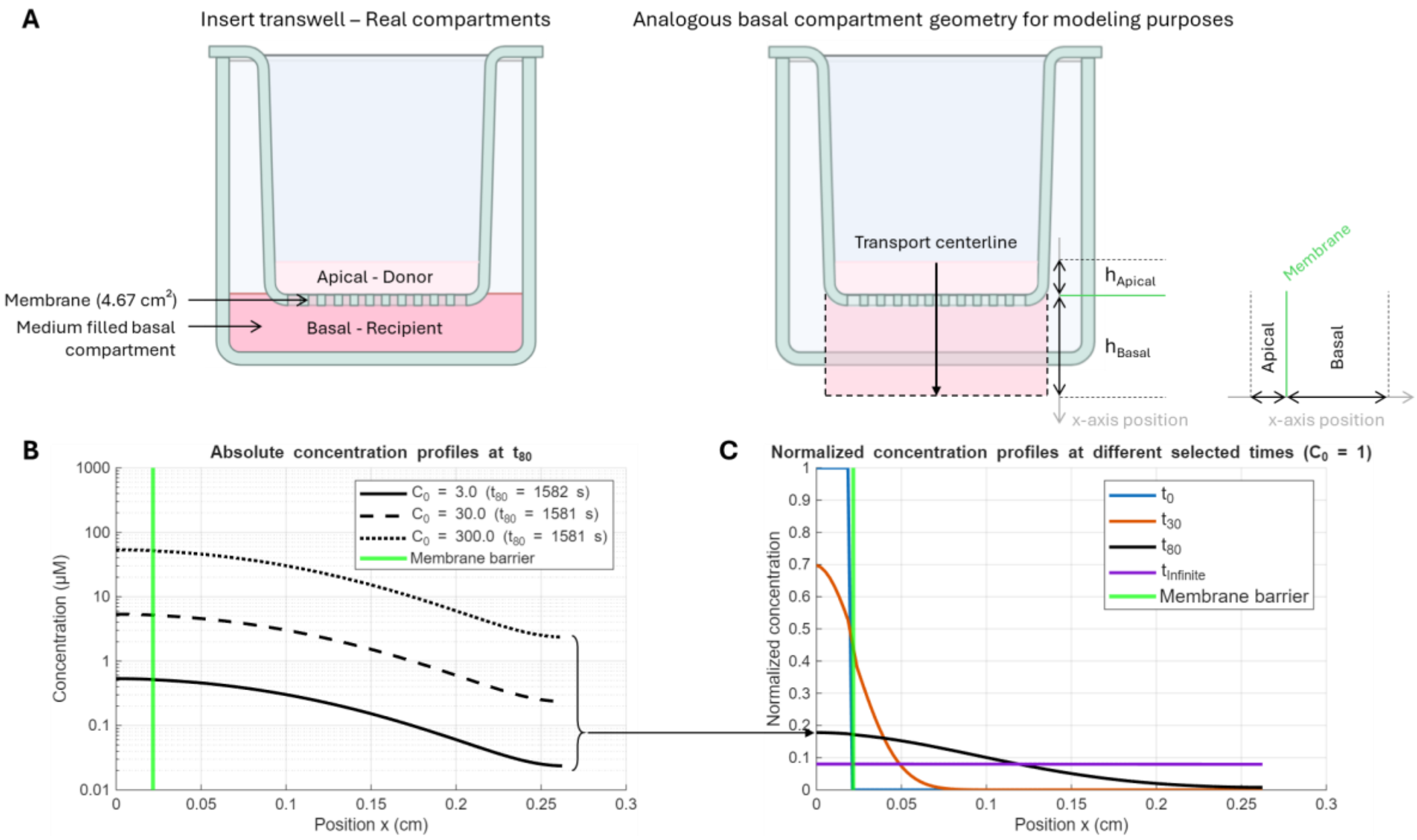

Supplementary Figure S6: *In silico* modelling of ASO transport across the membrane barrier model. (A) Schematic depiction of the geometric assumptions made for solving the 1-dimensional (1D) diffusion transport equation. The apical compartment is defined by the liquid layer deposited during pipetting application (100 µL) or nebulization (7 µL). The height of the apical compartment (h_{Apical}) is equal to 0.21 mm and 0.015 mm in pipetting and nebulization condition, respectively. The height of the 1D analogous basal compartment (h_{Basal} = 2.4 mm), was calculated from the applied medium volume and the geometry of the basal compartment. **(B)** The absolute concentration profiles of ASO (µM) for pipetting application at t_{80} (time at which 80% of the total dose has reached the basal compartment) as a function of x (position along the centerline of the transwell insert setup; see panel A) was obtained for three different initial ASO concentrations (C_0) of 3, 30 (as tested in our pipetting setting) and 300 µM. The parallel trend of the concentration profiles is shifted by the ratio of the respective C_0 values. This can be concluded from the fact that t_{80} of 1581s is independent of C_0. Thus, one can derive a characteristic (normalized) concentration profile for each time point (t_{10}, t_{20}, etc.) which is characteristic for the geometry of the setup (here: only h_{Apical} varies for pipetting application or nebulization). This is confirmed by the black line in panel C. **(C)** Normalized concentration profiles of ASO at different selected times (t_0, t_{30}, t_{80}, and $t_{Infinite}$) for pipetting application. For this, the absolute concentration profile (in µM) was normalized to the respective C_0. When applying this normalization process, all concentration profiles for t_{80} presented in panel B collapse to the same profile (black line) emphasizing that shape of C(x) varies with time and geometry, but not with C_0. In other words, if the basal concentration is presented as dose relative to the initial apical dose D_0 (%D), the theoretically expected time point for each of these values can be compared with the measured time point.

Reference:

[1] M. Strunz *et al.*, "Alveolar regeneration through a Krt8+ transitional stem cell state that persists in human lung fibrosis," *Nat. Commun.*, vol. 11, no. 1, 2020, doi: 10.1038/s41467-020-17358-3.

[2] H. Ren, N. P. Birch, and V. Suresh, "An optimised human cell culture model for alveolar epithelial transport," *PLoS One*, vol. 11, no. 10, 2016, doi: 10.1371/journal.pone.0165225.
